# Selected Ion Channels in Chemotherapy-Induced Peripheral Neuropathy: Mechanisms and Translational Limits

**DOI:** 10.3390/biom16101502

**Published:** 2026-10-15

**Authors:** Xunlang Zhang, Dekun Liu, Mingkai Yu, Ruibin Zhang, Lingling Liu, Jinyan Liu, Xiaojing Liu, Yingjie Wang, Shuguang Yu, Sha Yang

**Affiliations:** Acupuncture and Tuina School, Chengdu University of Traditional Chinese Medicine, No. 37, Shierqiao Road, Chengdu 610075, China

**Keywords:** chemotherapy-induced peripheral neuropathy, ion channels, sensory excitability, channel modulation, axonal injury, translational pharmacology

## Abstract

Chemotherapy-induced peripheral neuropathy (CIPN) combines abnormal sensory firing with neuronal and axonal injury. This critical narrative review focuses on selected voltage-gated sodium, calcium, and Kv7 potassium channels and transient receptor potential channels in chemotherapy-specific systems and human studies. Acute modulation is distinguished from altered channel abundance or localization and persistent remodeling. Human axonal-excitability studies support membrane dysfunction during acute oxaliplatin neurotoxicity but do not identify a sodium-channel subtype. Experimental studies identify changes in trafficking, stimulus sensitivity, and current that vary by time and compartment. Clinical analgesia does not itself demonstrate channel engagement or axonal protection. Duloxetine provides a symptomatic benchmark; results for channel-directed drugs and cannabinoid formulations remain inconsistent or design-limited. Natural products are treated as defined pharmacological examples, not as ranked candidates. A six-domain reporting template links target identity, potency, exposure, selectivity, CIPN context, and safety without assigning a composite score or evidence grade. Translation requires matching channel pharmacology to achievable unbound exposure and testing symptom relief separately from preservation of axons and protective sensation.

## 1. Introduction

Chemotherapy-induced peripheral neuropathy (CIPN) can present as burning pain, tingling, and stimulus-evoked hypersensitivity, as well as numbness, proprioceptive loss, and impaired balance. Platinum compounds, taxanes, vinca alkaloids, and proteasome inhibitors produce overlapping syndromes through partly different mechanisms. The chemotherapy agent, cumulative exposure, time since treatment, and outcome instrument all affect the apparent prevalence and clinical course [1,2,3]. This heterogeneity has a mechanistic consequence: an explanation for acute cold-evoked symptoms after oxaliplatin cannot be assumed to explain persistent sensory loss after paclitaxel.

This distinction has direct therapeutic implications. The 2020 American Society of Clinical Oncology (ASCO) guideline supports duloxetine for established painful CIPN while describing its average benefit as limited; it does not recommend a pharmacological agent for prevention [4]. Pain relief, recovery of sensation, and preservation of axons are different claims. A treatment can reduce firing without preventing injury, and excessive suppression of normal conduction may worsen sensory function. The relevant objective for a channel-directed analgesic is therefore to reduce pathological excitability while retaining useful afferent signaling, rather than to suppress peripheral conduction indiscriminately.

Human threshold-tracking studies provide a basis for channel hypotheses in acute oxaliplatin neurotoxicity [5,6]. We interpret each finding in relation to the chemotherapy agent, sampling time, compartment, assay, and perturbation used. The mechanistic examples focus on paclitaxel and oxaliplatin and on selected voltage-gated sodium (NaV), calcium (CaV), transient receptor potential (TRP), and neuronal Kv7/KCNQ channels; extrapolation to other agents is identified explicitly. Comparisons address why observations may differ and how formulation, route, and exposure constrain the link from channel pharmacology to intervention. This is not comprehensive coverage of CIPN channels or treatments.

We chose a critical narrative synthesis because human axonal physiology, channel experiments, intervention trials, and product pharmacology address different links in a causal argument. These evidence types do not yield one pooled treatment effect. Narrower questions about a channel, drug, or outcome remain suitable for systematic synthesis. We distinguish directly measured changes, same-system perturbation evidence, and extrapolations.

## 2. Review Scope and Methods

We selected the literature purposively; this was not a systematic or scoping review. It was not prospectively registered and did not use dual-independent screening, a prospectively specified stopping rule, or an exhaustive intervention denominator. The Scale for the Assessment of Narrative Review Articles (SANRA) informed clarity of scope and scientific reasoning, but it was not used as a substitute for transparent retrieval methods or study-level appraisal [7]. Exact return counts and the earlier named-entity distribution are not used to infer literature coverage, comparative evidence strength, or the frequency of evidence gaps.

The targeted search was updated through 30 September 2026. Europe PMC title/abstract queries and a corresponding PubMed query combined chemotherapy-associated neuropathy with ion-channel or excitability terms. Queries were run in English, and no formal translation workflow was used; non-English evidence may therefore be underrepresented. Older primary studies were also checked through citation tracing and named-target searches where a reviewer-identified gap required clarification. Supplementary Table S1 and Data S1 retain the exact dated update queries, returned identifiers, selection decisions, and source-access limitations. The earlier retrieval archive remains a historical record rather than a systematic screening denominator. The update does not establish exhaustive coverage or retrospectively repair incomplete historical search logs.

**Figure 1:**
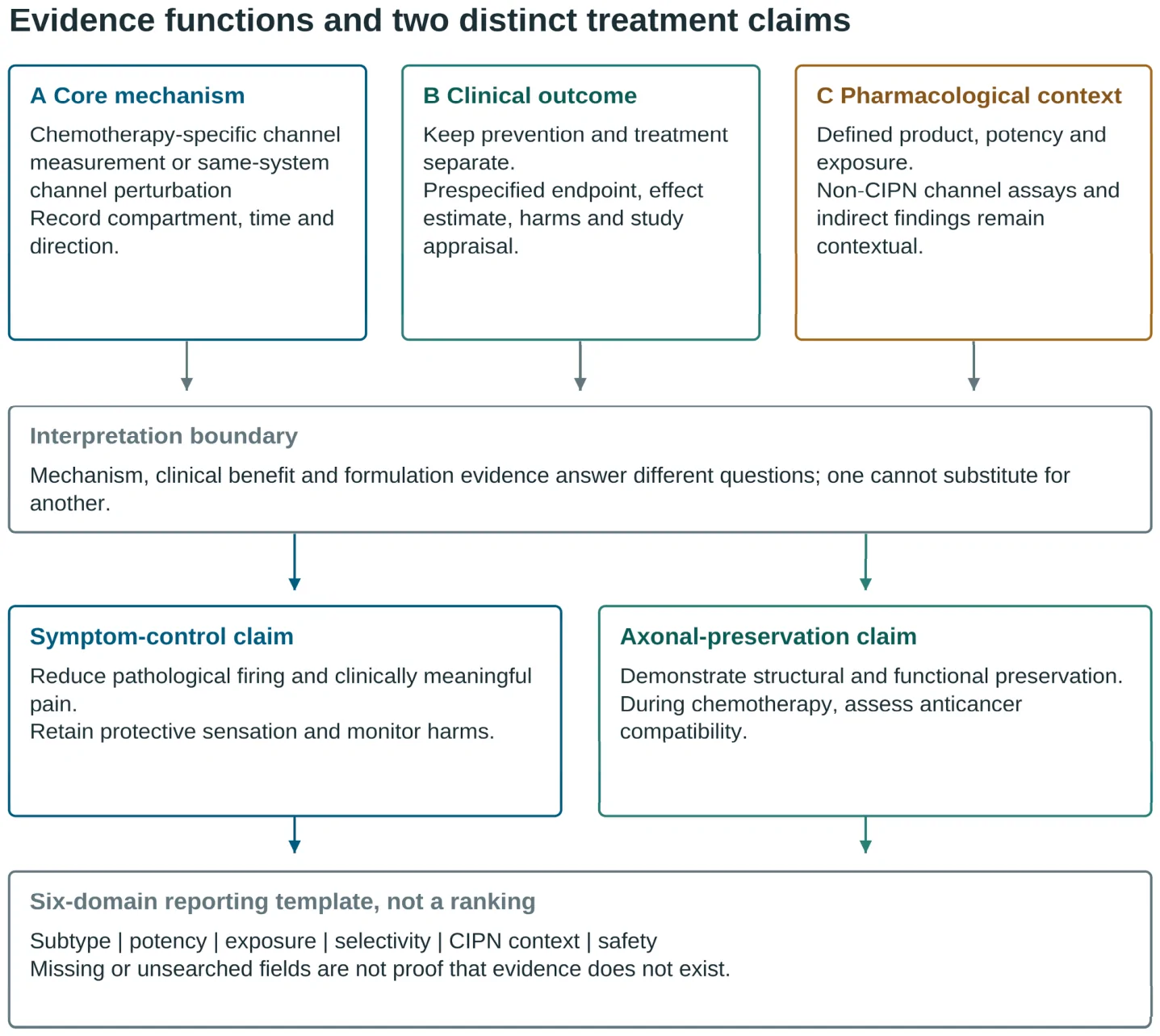
Evidence functions and distinct treatment claims. Mechanistic, clinical, and pharmacological evidence are not interchangeable. The six-domain template organizes reporting without scoring or ranking products.

A core mechanism claim was required to show a chemotherapy-specific observation of channel function, expression, or localization; a channel-directed perturbation in the same CIPN system; or direct human target-engagement evidence. These routes address different levels of evidence and are not interchangeable. A change in transcript or protein abundance is a molecular association unless accompanied by functional or causal tests. A channel-resolved assay outside CIPN defines pharmacology but does not establish a CIPN mechanism. Antioxidant effects, cytokine changes, network-pharmacology predictions, and behavioral improvement alone do not meet the core channel-mechanism threshold. Such findings are restricted to contextual examples rather than entered into the core mechanism matrix. Figure 1 summarizes these evidence functions and the distinct symptom-control and axonal-preservation claims.

For each core mechanism claim, the records specify species or human population, chemotherapy, exposure or assessment time, anatomical compartment, measurement, direction of change, and perturbation evidence. The detailed records and source-access status are provided in Supplementary Table S2. Clinical interpretation separates prevention from treatment, painful from predominantly sensory-loss phenotypes, randomized from non-randomized designs, and prespecified from exploratory outcomes. Supplementary Table S3 identifies the primary endpoint; analysis population; time point; absolute results; between-group estimate; confidence interval, where reported; harms; and unresolved reporting limitations. Abstract-only material does not anchor efficacy conclusions.

We performed a single source audit of selected clinical results using the revised Cochrane risk-of-bias tool for randomized trials (RoB 2). For the uncontrolled QUCIP study, we used the Joanna Briggs Institute (JBI) quasi-experimental checklist, 2020 version [8,9,10]. First-period comparisons were assessed with the parallel-group tool, whereas a contrast using both periods was assessed with the crossover version. Original reports, available protocols, figures, and registry records were checked against each item. Supplementary Table S4 and Data S1 identify the exact result, tool version, source passages, answers, and algorithm outputs. Unresolved information remains explicit, and an uncertainty-driven algorithm output is not presented as proof of bias. These result-specific assessments are not certainty grades or product rankings. Because QUCIP is a single-arm before–after observation without a comparator, the JBI checklist was used for structured methodological description. ROBINS-I was not applied to this non-comparative report, and we did not attempt a cross-study causal risk-of-bias ranking.

The selected clinical evidence table described below is a concise overview rather than an all-study risk-of-bias table. Only rows with a corresponding worksheet in Supplementary Table S4 are formally appraised; rows marked context-only are reported descriptively and are not used for cross-study causal comparisons.

We use the six-domain template only as a reporting aid; it is not a composite score, confidence grade, or candidate-ranking method. Its rationale draws on Minimum Information About a Bioactive Entity (MIABE), exposure–target–pharmacology principles, and the 5R drug-development framework [11,12,13]. It asks whether the same identified product can be linked across target, potency, exposure, selectivity, CIPN context, and safety. We distinguish a field not reported in a consulted source from a field not examined in the present retrieval and a result not identified within a specified search. The label “none” is reserved for cases in which the relevant evidence was explicitly assessed and absent; otherwise, the field is coded as not reported, not searched, or not identified. Representative worked examples are provided in the corresponding main-text table and Supplementary Tables S5 and S7; botanical identity requirements are recorded separately in Supplementary Table S6.

## 3. Chemotherapy-Specific Channel Mechanisms

### 3.1. Acute Modulation and Persistent Remodeling

Here, acute modulation means a change in channel gating or current over the observed exposure period; it need not involve altered channel abundance. Remodeling is used more narrowly for changes in expression, membrane localization, or organization, and persistence is claimed only when demonstrated over a defined observation or washout period. These processes may occur together, but a rapid change in excitability is not, by itself, evidence of transcriptional remodeling. Likewise, a transcriptomic signal does not establish the direction of functional current. Table 1A summarizes the selected chemotherapy–channel–phenotype relationships, with the detailed measurements retained in Supplementary Table S2. Table 1B provides one shared summary of modulators, evidence types, and human translational limits for Section 3.2, Section 3.3, Section 3.4 and Section 3.5.

Anatomical compartment is part of the mechanistic claim. A dorsal root ganglion (DRG) somal measurement does not establish distal-axon function, and spinal astrocyte calcium signaling is not peripheral nociceptor engagement. We distinguish peripheral axons, DRG neurons, satellite glia, spinal astrocytes, and central sensory synapses; findings in one compartment motivate, but do not substitute for, measurements in another.

The selected studies primarily address hypersensitivity and excitability. Table 1 does not assign a validated channel subtype to numbness, proprioceptive loss, or sensory ataxia. For these loss-of-function phenotypes, structural injury and functional measures remain necessary; a channel association in a painful model cannot fill that gap.

Acute oxaliplatin symptoms offer a human physiological example. Recovery-cycle and threshold-electrotonus abnormalities are compatible with altered axonal sodium-channel kinetics [5,6]. Threshold tracking measures the behavior of an axon population, however, rather than an isolated channel current. It does not identify NaV1.6, NaV1.7, or NaV1.8 as the molecular mediator. These observations should be described as human axonal-excitability evidence, not direct subtype engagement. Their relevance to later sensory loss also requires longitudinal testing rather than an assumption that the acute and chronic syndromes share one initiating mechanism.

**Figure 2:**
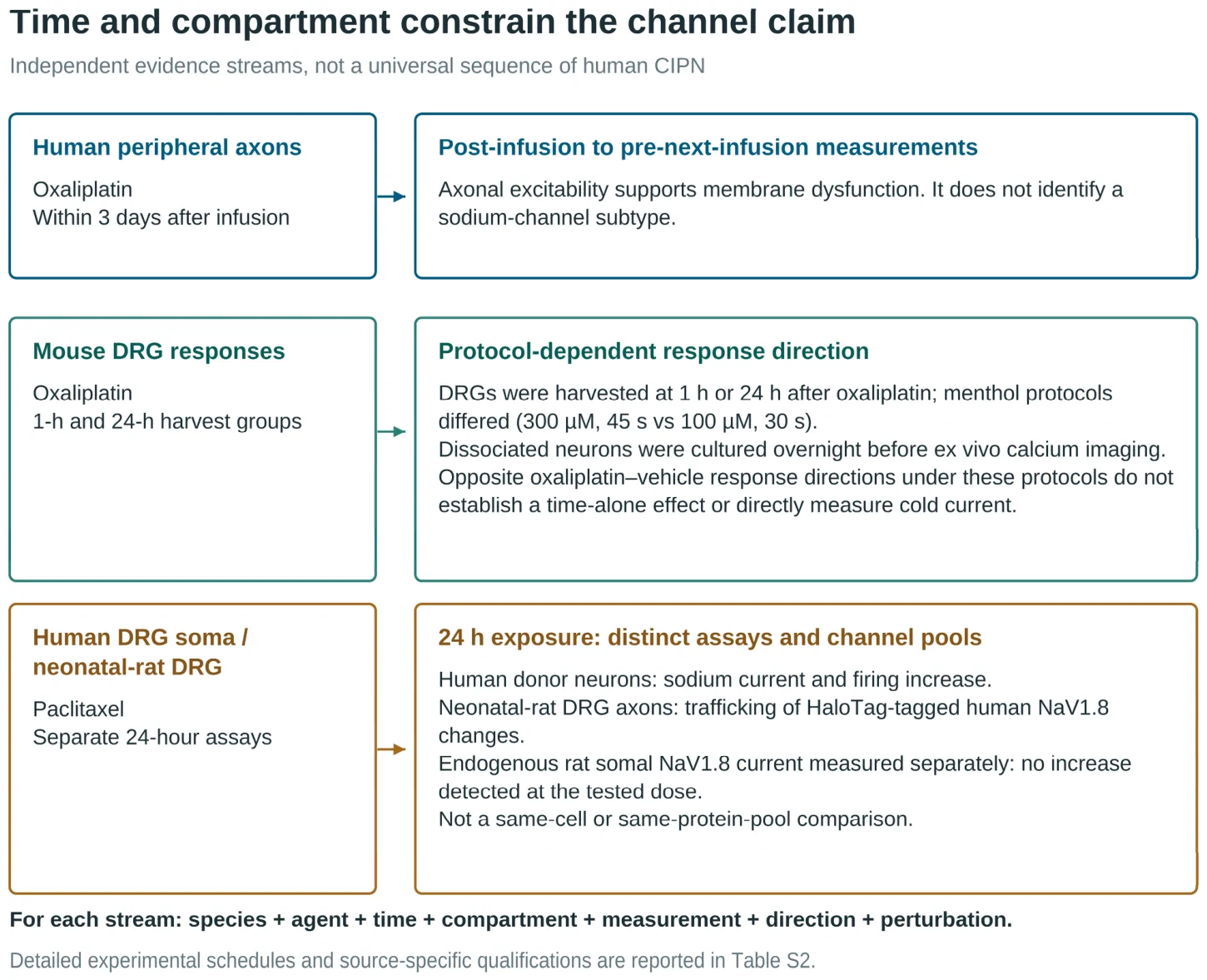
Parallel study-specific temporal and compartmental observations. Patient oxaliplatin recordings [6], mouse TRPM8 harvest groups [18], human DRG sodium responses [15], and NaV1.8 trafficking in rat neurons [16] have different time origins and preparations. Mouse DRGs were harvested at 1 or 24 h and cultured overnight before calcium imaging. The menthol protocols differed (300 µM/45 s versus 100 µM/30 s), so these comparisons do not isolate harvest time. Tagged human NaV1.8 trafficking and endogenous rat somal current were measured separately. No common disease clock, paired endogenous channel pool, or clinical subtype engagement is implied.

Figure 2 compares study-specific observations across time and measurement settings. Oxaliplatin-treated mouse DRG harvest groups showed opposite menthol-response directions relative to their respective vehicle controls after overnight culture. Menthol concentration and stimulation duration also differed, so time alone cannot explain the contrast. Paclitaxel-treated rat neurons showed increased distal labeling of tagged human NaV1.8 but unchanged separately measured endogenous somal current, which does not establish different currents across compartments. Matched localization and subtype-resolved current measurements, with defined exposure and washout windows, would test that functional link. Neither comparison establishes progression to chronic human axonal loss (Section 3.2 and Section 3.4).

### 3.2. Sodium Channels

Voltage-gated sodium channels generate and propagate action potentials, but individual subtypes contribute differently to threshold, repetitive firing, and conduction. In cultured human DRG neurons, 24 h paclitaxel exposure increased *SCN9A* expression, transient sodium current, and firing; the measured current was not an isolated NaV1.7 current [15]. In an oxaliplatin mouse model, *Scn8a* transcript and NaV1.6 protein increased, whereas the measured NaV1.6 current showed only an upward trend relative to vehicle. TET1 overexpression reduced that current relative to the oxaliplatin control and attenuated hypersensitivity, supporting an upstream regulatory mechanism rather than proving a significant chemotherapy-induced current increase [14]. These results come from different systems and levels of specificity. They do not establish that all painful CIPN is a NaV1.7 disorder or that acute human oxaliplatin signatures resolve to NaV1.6. A longitudinal human study of 58 patients receiving oxaliplatin (FOLFOX4/6 or XELOX) included 905 axonal-excitability assessments. Acute sensory recovery-cycle changes and later cumulative sensory abnormalities (reduced refractoriness, increased superexcitability, and altered 90–100 ms threshold electrotonus) preceded sensory response-amplitude loss; early-cycle excitability abnormalities predicted the final clinical outcome in 80% of the analyzed subgroup. This observational threshold-tracking result is compatible with a sodium-channel functional channelopathy, but it does not identify a NaV isoform or establish patient-level target engagement or causality [5].

Trafficking provides a second layer of regulation. In neonatal rat DRG cultures, paclitaxel increased distal delivery of HaloTag-tagged human NaV1.8. Endogenous rat NaV1.8 somal current density, measured separately, was unchanged in the 25 nM comparison [16]. These are different channel preparations, not paired axonal and somatic measurements of the same endogenous pool. The study supports attention to compartment and assay identity, without establishing increased endogenous axonal current. Translational experiments should combine localization with functional measurements in the same relevant system.

Subtype selectivity matters because physiological conduction and off-target cardiac or central nervous system effects constrain broad sodium-channel blockade. Suzetrigine illustrates pharmacological tractability; it does not provide evidence of CIPN efficacy. The US Food and Drug Administration (FDA) label specifies moderate-to-severe acute pain in adults, including postoperative pain, not CIPN [26]. A primary pharmacology study reported a human NaV1.8 half-maximal inhibitory concentration of 0.68 nM and strong selectivity over other human sodium-channel subtypes [25]. The marked species dependence of potency in that report cautions against importing a human potency threshold into a rodent experiment. Neither acute-pain approval nor analgesia in another indication establishes benefit in CIPN, preservation of protective sensation during chemotherapy, or prevention of axonal degeneration.

### 3.3. Calcium Channels and Their Auxiliary Subunits

Calcium-channel hypotheses involve both intrinsic excitability and synaptic transmission. CaV2.2 at sensory terminals contributes to transmitter release; α2δ subunits influence channel trafficking and are pharmacological targets of gabapentinoids. These mechanisms are established beyond CIPN, but a negative or positive gabapentinoid trial is not a direct assay of channel remodeling in chemotherapy-exposed human nerves [35,36]. The pore-forming channel and its auxiliary subunit should therefore not be used as synonymous target labels.

Paclitaxel-associated increases in T-type calcium current were measured in rat DRG neurons. In human DRG preparations, paclitaxel-sensitive inward current and firing were sensitive to ML218; these observations should not be described as an isolated human CaV3.2 current. In the animal study, early ML218 treatment attenuated development of hypersensitivity, whereas treatment at the tested established-neuropathy stage did not reverse it [22]. ML218 pharmacology does not establish exclusive CaV3.2 mediation. Human donor-tissue responses add biological relevance but are not engagement measurements in treated patients. Additional abstract-limited pathway reports are retained as contextual records in Table S2, without assigning unverified species, dose, or sampling intervals to the main mechanism matrix.

### 3.4. TRP Channels and Stimulus-Dependent Symptoms

Transient receptor potential channels connect chemical or thermal stimuli to depolarization. Oxaliplatin models support roles for TRPA1 and TRPM8 in cold-sensitive phenotypes through functional assays and pharmacological or genetic perturbation [17,18,19]. In one mouse study, DRGs were harvested 1 or 24 h after oxaliplatin and cultured overnight before ex vivo calcium imaging. Menthol-evoked responses increased in the 1 h harvest group and decreased in the 24 h group relative to their respective vehicle controls [18]. The groups received different menthol protocols (300 µM for 45 s and 100 µM for 30 s, respectively), so the contrast does not isolate a time effect. These are harvest-defined groups, not in vivo recordings at those times. An agonist-evoked calcium signal is not identical to a cold-gated current. These observations support agent- and time-specific hypotheses, not a single TRP mechanism for all platinum drugs or stages of neuropathy.

In a paclitaxel rat model, TRPV1 and TRPM8 changed in opposite directions: TRPV1 expression and agonist-evoked responses increased, whereas TRPM8 measures decreased. TRPV1 antagonism and TRPM8 agonism each attenuated mechanical hypersensitivity [20]. This is incompatible with a generalized diagram showing all TRP channels as upregulated. A reduction in an inflammatory marker followed by less pain behavior cannot by itself show that TRPV1 mediated the improvement; the relevant channel relation must be tested in the same system.

TRP pharmacology also challenges the assumption that inhibition is always therapeutic and activation always harmful. Capsaicin activates TRPV1 and can subsequently produce local terminal defunctionalization; this mechanism should not be called axonal protection [29]. Menthol activates TRPM8, but its consequences depend on concentration, delivery, and the underlying sensory state [32]. A cooling sensation in one setting cannot justify use in acute oxaliplatin cold hypersensitivity. Clinical formulation and local exposure are therefore integral to the direction of the proposed effect.

### 3.5. Kv7 and the Limits of the Selected Channel Scope

Kv7 findings distinguish acute current inhibition from pre-existing genetic loss. Paclitaxel inhibited KCNQ currents in rat sensory neurons and recombinant channels, whereas Advillin-Cre-mediated sensory-neuron *Kcnq2* deletion attenuated paclitaxel-associated hypersensitivity and fiber loss in mice [23]. Higher baseline fiber density makes compensation plausible, not established. In male rats, early retigabine attenuated behavioral and structural changes despite preserved tested *Kcnq2*, *Kcnq3*, and *Kcnq5* transcripts and KCNQ5 protein [24]. Preserved abundance does not exclude functional inhibition; protection after deletion does not establish therapeutic blockade. A discriminating experiment would compare non-inducible and adult-inducible sensory-neuron deletion under the same paclitaxel schedule, with baseline fiber measurements and channel re-expression controls. Current, excitability and fiber loss would be measured separately. Protection after adult deletion without baseline structural differences would weaken a developmental-compensation explanation; restoration of paclitaxel-evoked acute depolarization after re-expression would support channel dependence. These are proposed tests, not observed results. An author-supplied full-text rat/human sural-nerve study applied oxaliplatin at 10–30 µM (30 µM in the standard rat protocol) for 60 min and used flupirtine at 10 µM in rat nerve and 3–30 µM in human fascicles. Cooling-enhanced A-fiber after-activity was reduced by flupirtine, whereas the experiment did not establish a selective Kv7 isoform current or clinical analgesic efficacy. The result supports acute ex vivo enhancement of a slow potassium conductance, consistent with Kv7-related pharmacology, rather than in vivo treatment benefit [33].

A recent review integrates channel biology, disease models, and translational strategies [37]; this selected comparison complements its broader scope by examining the conditions under which each finding supports a mechanistic claim. New patient-linked evidence further refines the TRPV1–paclitaxel hypothesis: transient 18:1 lysophospholipid elevations after dosing activated TRPV1 in sensory-neuron and heterologous-channel assays, but the observational cohort did not establish patient-level mediation, direct patient target engagement, or chronic axonal remodeling [21]. Adenosine triphosphate-gated P2X receptors, acid-sensing ion channels, mechanosensitive Piezo channels, two-pore-domain potassium channels, and connexin or pannexin channels address additional neuronal and non-neuronal processes. Incomplete retrieval does not establish their irrelevance. Table S2 provides boundary examples, not exhaustive coverage. Hyperpolarization-activated cyclic nucleotide-gated (HCN) and Kv4.3 findings likewise require chemotherapy- and compartment-specific interpretation [38,39]. A full-text study provides a non-neuronal boundary example outside the core neuronal map: paclitaxel (8 mg/kg intraperitoneally, four injections at 2-day intervals) produced mechanical hypersensitivity in mice from 2 days through 3 weeks. Optogenetic or chemogenetic keratinocyte inhibition and epidermal Piezo1 deletion reduced this behavior. Paclitaxel (1 µM, 30 min) also increased mechanically evoked and Yoda1-evoked responses in mouse and healthy-donor human keratinocytes and increased PIEZO1 current amplitude/conductance in PIEZO1-expressing HEK293T cells without evidence of increased channel expression. This is preclinical epidermal evidence without patient target engagement and remains a scope-boundary mechanism rather than a core neuronal channel conclusion [40]. A vincristine study is retained as a central-astroglial boundary example: adult male ICR mice receiving vincristine 0.1 mg/kg intraperitoneally for 5 consecutive days and primary neonatal cortical astrocytes exposed to 3 nM vincristine showed increased spinal GFAP, phosphorylated CaMKII, CaV3.2/*CACNA1H* and Cx43, altered Fluo-4 calcium signals and inflammatory readouts; intrathecal KN-93 or L-ascorbate and downstream Cx43 perturbation with Gap27 or siRNA attenuated short-latency hypersensitivity and cytokine release [41]. Because it lacked CaV3.2 current/gating measurements, genetic *CACNA1H* loss/rescue, and patient target-engagement data, we classify it as an indirect spinal-astrocyte pathway signal rather than channel-resolved CaV3.2 evidence or evidence of translational readiness. The boundary records include a paclitaxel–NaV1.7/ERK association [42], oxaliplatin-related ion-channel expression remodeling [43], a P2X7/CGRP preclinical signal [44], an endothelial/non-neuronal Piezo2 boundary example in an oxaliplatin model [45], a negative pannexin-1 CIPN study [46], and the recent LEP-4, NCX2 and dorsal-horn METTL3/circMtmr7 reports [47,48,49]. These records remain contextual and do not expand the core channel-resolved map.

### 3.6. Excitability Control and Axonal Preservation

Channel-directed symptom control and axonal preservation are separate development objectives. The former aims to reduce pathological firing while retaining normal sensation. The latter requires evidence that an intervention prevents or limits structural degeneration and functional loss. Pain relief alone establishes neither preserved axon number nor recovered proprioception. Conversely, a structural effect without a meaningful functional benefit may not justify a disease-modification claim. The two objectives can be tested in the same program but should have separate endpoints and conclusions.

Sterile alpha and TIR motif-containing protein 1 (SARM1) illustrates the preservation axis without expanding the core channel map. Genetic perturbation in human induced pluripotent stem cell-derived sensory neurons supports a role in vincristine-associated axon degeneration, while paclitaxel models provide additional preservation evidence [50,51]. These results concern an axon-degeneration pathway rather than direct channel modulation. They motivate combined measurements of axonal structure, conduction and sensory function, but do not establish a clinically effective neuroprotective treatment. Upstream immune or mitochondrial interventions belong in this context unless the same experiment also demonstrates a channel-mediated effect.

## 4. Clinical Evidence Tests Different Questions

### 4.1. Treatment of Established Painful CIPN

Clinical benefit and mechanistic validation require separate evaluation. A trial can establish improvement in pain without measuring channel engagement, and a ligand with well-characterized pharmacology can fail clinically because of exposure, phenotype, tolerability or an incorrect disease mechanism. Table 2 provides a concise clinical comparison; the source-specific endpoint and appraisal records are in Supplementary Tables S3 and S4. We do not assign informal confidence grades or use these comparisons to generate an individual prescribing algorithm.

Duloxetine provides a symptomatic benchmark rather than evidence of ion-channel engagement. In the trial by Smith et al., 231 participants were randomized and 220 began treatment. The verified first-period primary analysis included 181 observed outcomes (87 duloxetine-first and 94 placebo-first), distinct from randomized, treatment-starter, and imputed populations. At 5 weeks, average pain decreased by 1.06 points with duloxetine and 0.34 with placebo; the between-group advantage was 0.73 points on the 0–10 scale (95% confidence interval [CI], 0.26–1.20). The supplied eAppendix specifies 100 MCMC-based multiple-imputation datasets followed by regression imputation and a control-based pattern-mixture analysis. The imputed between-group differences were 0.750 (*p* = 0.0015) and 0.667 (*p* = 0.0037), respectively; the reported proportions of imputed values were 7.1%, 10.8%, 13.7%, 14.6%, and 14.6% for weeks 2–6. Exploratory relative risks for at least 30% and 50% pain reduction were 1.96 (95% CI, 1.15–3.35) and 2.43 (95% CI, 1.11–5.30), respectively. Pain-interference improvement favored duloxetine by 4.40 points on the 0–70 summed scale (95% CI, 0.93–7.88). The main article reports adverse-event-related discontinuation of 11% versus 1%; the eAppendix additionally lists grade 2–3 adverse events by initial-treatment arm, but these exposure tables should not be used to reconstruct discontinuation numerators. These results concern short-term symptom treatment, not prevention or axonal restoration.

Gabapentin did not demonstrate improvement in the prespecified pain or neuropathy outcomes in the 115-participant N00C3 crossover trial [27]. Its population was not uniformly enriched for pain, because eligibility could also be met through neuropathy symptoms. In the smaller pregabalin crossover study, the primary hypothesis tested whether mechanical pain sensitivity predicted response, not analgesic superiority. That hypothesis was not supported; the secondary average-pain comparison was also not significant, and a favorable oxaliplatin subgroup result was post hoc [28]. Dizziness, somnolence, treatment identification, and missing outcomes further constrain interpretation. A separate randomized active-comparator phase II study in 82 breast-cancer patients with established taxane neuropathy reported greater six-week improvement with pregabalin than duloxetine across VAS, NCI-CTCAE, and PNQ outcomes, but it had no placebo arm and used a single breast-cancer center [52]. Its published adjusted GEE estimates are retained as source-reported contrasts, not as placebo-controlled effects. These studies address different questions and do not establish equivalence or a cross-population treatment hierarchy.

The clinical section is intentionally selective. Additional sodium-channel agents and compounded topical products discussed in the submitted version are not used here to build a comparative efficacy hierarchy where the necessary full-text and result-level appraisal could not be completed. Their pharmacological plausibility cannot fill that evidentiary gap. A negative or inconclusive intervention study would also not, by itself, establish that a molecular subtype is irrelevant to CIPN.

High-concentration capsaicin is a formulation-specific local intervention. Its active ingredient has a clear TRPV1 mechanism. The selected QUCIP report is an uncontrolled observational study and cannot distinguish treatment effect from natural history, co-interventions, or expectation [30]. A reduction in a symptom score after treatment is not a randomized efficacy estimate. The 8% patch should remain distinct from low-concentration cream and from unrelated topical formulations. Likewise, terminal defunctionalization may reduce pain but is not evidence of axon rescue. Local tolerability and sensory function require assessment in addition to pain.

### 4.2. Prevention Is Not Treatment

A preventive intervention is given before or during evolving injury and must be tested against incident or worsening neuropathy in a specified chemotherapy context. A treatment trial recruits patients with an established phenotype. The populations, endpoints, and competing risks differ. Acute symptom relief during oxaliplatin treatment should not be described as prevention of chronic neuropathy unless the trial actually measured that outcome. These distinctions also prevent a favorable result with one taxane regimen from being generalized to platinum-associated sensory neuronopathy.

Venlafaxine illustrates the distinction between acute symptoms and prevention. The full EFFOX report [53] describes patients with established acute oxaliplatin neurotoxicity randomized to venlafaxine (50 mg 1 h before infusion, and then extended-release 37.5 mg twice daily on days 2–11) or placebo. Forty-eight patients were randomized (24/24), with 20 venlafaxine and 22 placebo patients assessable for neurotoxicity. Reported complete acute relief was 31.3% versus 5.3% (*p* = 0.03), and relief of at least 50% was 68.8% versus 26.3% (*p* = 0.02); no confidence intervals were reported. Pins-and-needles and functional impairment favored venlafaxine, whereas cold-triggered pain was not conventionally significant. The trial stopped early, and published percentages and adverse-event denominators do not map cleanly to the randomized or assessable denominators; event counts and CIs were therefore not reconstructed. This study addresses acute symptom relief, not chronic CIPN prevention or axonal preservation. In the subsequent MC11C4 prevention pilot, the longitudinal sensory outcome did not favor venlafaxine (*p* = 0.55) [54].

Indirect interventions can provide counterexamples without occupying a parallel catalog. In SWOG S0715, acetyl-L-carnitine did not improve the prespecified 12-week neuropathy endpoint (adjusted difference, −0.91; 95% CI, −2.21 to 0.39; *p* = 0.17). The secondary 24-week Functional Assessment of Cancer Therapy neurotoxicity (FACT-NTX) comparison was worse with acetyl-L-carnitine (−1.80; 95% CI, −3.16 to −0.43; *p* = 0.01; higher scores indicate fewer symptoms) [55]. These different time points should remain distinct, and the later result should not be relabeled a prespecified primary outcome. The example illustrates why biological plausibility is insufficient to justify prevention; it is not evidence of direct channel engagement. The Alliance N0992 compliance analysis [56], the A221805 duloxetine prevention trial [57], and the SWOG S0715 two-year follow-up [58] are retained as protocol/completeness and longitudinal-context sources rather than as additional independent efficacy estimates.

### 4.3. Cannabinoid Formulations Are Not Interchangeable

A placebo-controlled topical cannabidiol (CBD) pilot did not demonstrate benefit for its first-period sensory endpoint. Although 40 participants were randomized and 38 appeared in the overall analysis, the original figures identify only 35 in the week-2 primary analysis (19 CBD-first and 16 placebo-first); no primary between-group estimate with a CI was reported in the inspected article [59]. Haney et al. reported an 8-week double-blind pilot in 12 women with painful taxane neuropathy (6/6 randomized) using oral CBD 100 mg plus THC 5 mg up to three times daily; all participants completed the study, but dose reductions were common. Pain and several function measures improved over time in both groups, without a treatment-specific pain benefit; active cannabis was associated with worse neuropathy ratings (*p* = 0.035), lower functional well-being in weeks 6–8 (all *p* < 0.02), and greater sleep disturbance (all *p* < 0.05), with no severe adverse events. The study was underpowered and exploratory, and individual BPI pain-interference contrasts were not significant (all *p* > 0.12) [60]. In a 12-week oral CBD/tetrahydrocannabinol (THC) trial, the primary sensory-score change comparison was not significant (*p* = 0.24); the nominally favorable adjusted sensory estimate came from an exploratory model and was not accompanied by improved average pain [61]. The Nielsen et al. 2022 report [62], described in the article as CINCAN-2, provides a prospective, open-label, non-randomized early-prevention comparison. The public record located for CINCAN-2 is NCT04582591, whereas the article/PubMed display separately prints “NCT 04,167,319”; the identifiers were not reconciled and neither was used to replace the article’s reported data: 54 patients received pure oral CBD 150 mg twice daily for eight days, beginning one day before CAPOX or carboplatin–paclitaxel chemotherapy, and were compared with historical CINCAN-1 controls. Adjusted day-4 Dig-II 250-Hz vibrometry favored CBD in CAPOX (−1.22, 95% CI of −2.05 to −0.39) and carboplatin–paclitaxel (−1.76, 95% CI of −2.52 to −1.02), while patient-reported cold-sensitivity items favored CBD mainly in CAPOX and not in carboplatin–paclitaxel. Age, comorbidity, temperature, G-CSF, and missingness differed between cohorts, and the study was limited to acute first-cycle outcomes. It is hypothesis-generating and does not establish causal prevention or channel engagement. The three randomized/controlled analyses and the non-randomized cohort use different designs and analysis populations and should not be pooled or ranked [62].

The relevant unit is the product used in the trial: its cannabinoid composition, dose, route, duration, and intended use. Oral cannabidiol, cannabidiol/tetrahydrocannabinol combinations, an oromucosal extract, and a topical cream cannot be pooled conceptually merely because they share one ingredient. A channel assay on isolated cannabidiol is not a pharmacodynamic measurement of any of these clinical formulations. This distinction separates molecular plausibility from an exposure-supported mechanism in patients.

**Table 2:** Selected clinical findings separated by question and design.

Question/Phenotype	Product, Design and Population	Selected Result and Quantitative Limit	Main Interpretive Constraint
Treatment; painful CIPN	Duloxetine; first 5-week period; 231 randomized/220 treated/181 observed primary outcomes (87/94) [63]	Pain decrease of 1.06 vs. 0.34 points; advantage 0.73 (95% CI 0.26–1.20). Exploratory responder and functional results in text.	Article reports AE discontinuation 11% vs. 1%; conflicting flowchart counts retained in S3. Observed n is not an imputed-analysis n.
Treatment; symptomatic, not uniformly pain-enriched	Gabapentin; crossover; 115 randomized [27]	No demonstrated primary pain/neuropathy benefit. CI for contrast not reported in inspected source.	Incomplete follow-up; differing sensory/pain eligibility.
Treatment; painful CIPN	Pregabalin; crossover; 26 enrolled; 24 entered both periods. Exact published paired-analysis n unresolved [28]	Primary prediction hypothesis unsupported. Secondary pain decrease of 0.8 vs. 0.5 points; *p* = 0.08; CI not reported.	Flow, Table 2, Discussion and registry denominators differ. Unblinding and missingness; secondary and post hoc hypotheses distinguished.
Treatment; established sensory symptoms	Topical CBD cream; 40 randomized/38 overall analyzed/35 primary week-2 outcomes (19/16) [59]	No first-period sensory benefit demonstrated; primary between-group estimate/CI not reported in inspected article.	Original figures verify 19/16; registry 20/17 is a shared series denominator. No washout complicates second-period, not first-period, inference.
Treatment; grade 2–3 CIPN	Oral CBD/THC; randomized; 46 randomized/43 primary analysis [61]	12-week sensory improvement 13.5 vs. 9.3 points; *p* = 0.24. Exploratory adjusted estimate −10.4 (95% CI −20.5 to −0.3) is not the primary result.	Different models/analysis sets; multiplicity; average-pain comparison not favorable.
Prevention during oxaliplatin	Venlafaxine; randomized MC11C4 pilot; 50 accrued [54]	Longitudinal sensory endpoint *p* = 0.55; effect CI not reported in inspected text.	Pilot precision; not an established-pain treatment trial.
Observational treatment; painful CIPN	Capsaicin 8% patch; QUCIP; 73 treated; primary scores available for 72 at baseline and 56 at week 12 [30]	12-week painDETECT paired change −4.00 (SD 6.05); paired n not separately stated; no concurrent between-group effect or CI.	Uncontrolled; 35/73 discontinued over 36 weeks, not all before week 12. No causal efficacy claim.
Treatment; established taxane sensory neuropathy	Pregabalin versus duloxetine; randomized active-comparator phase II; breast cancer; 82 analyzed (40/42) [52]	Six-week VAS ≥ 33% improvement: 37/40 (92.5%) versus 16/42 (38.1%), *p* < 0.001; NCI-CTCAE sensory improvement: 37/40 versus 13/42 (31%), *p* < 0.001; PNQ improvement: 36/40 (90%) versus 13/42 (31%), *p* < 0.001. Published adjusted GEE estimates for duloxetine versus pregabalin were VAS + 4.71 (95% CI 2.30–7.13), NCI grade + 0.36 (0.21–0.51), and PNQ + 0.56 (0.38–0.75), all *p* < 0.001.	No placebo arm; single-center breast-cancer study; reported blinded allocation but active-drug masking and comparator-dose interpretation remain limitations. Results do not establish superiority to placebo or generalize to other cancers. Context-only; not formally appraised in S4; no cross-study causal ranking.
Prevention during paclitaxel; acute pain and evolving CIPN	Pregabalin 75 mg twice daily versus placebo; multicenter randomized pilot; weekly paclitaxel 80 mg/m^2^ for 12 weeks; 46 randomized (23/23), 41 first-cycle primary-evaluable (19/22) [64]	Cycle-1 maximum worst pain over the first six days: pregabalin 2.6 (SD 2.5) versus placebo 3.2 (SD 3.0), *p* = 0.56. EORTC QLQ-CIPN20 sensory growth-curve and AUC comparisons *p* = 0.88 and 0.46; no consistent tingling or pain benefit.	Pilot precision; no reported effect CI. Five participants lacked primary baseline data. Acute paclitaxel pain and longitudinal questionnaire findings do not establish chronic-CIPN prevention. Context-only; not formally appraised in S4; no cross-study causal ranking.
Acute oxaliplatin neurotoxicity; symptomatic relief	Venlafaxine 50 mg before infusion then extended-release 37.5 mg twice daily on days 2–11 versus placebo; randomized double-blind EFFOX trial; 48 randomized (24/24), 20/22 assessable [53]	Reported complete relief: 31.3% versus 5.3%, *p* = 0.03; ≥50% relief: 68.8% versus 26.3%, *p* = 0.02. Pins-and-needles and functional impairment favored venlafaxine (both *p* < 0.001); cold-triggered pain *p* = 0.06. No effect CI was reported.	Small trial stopped early. Published percentages and adverse-event denominators do not map cleanly to 20/22 or 24/24; event counts and CIs were not reconstructed. Acute relief is not chronic-CIPN prevention. Context-only; not formally appraised in S4; no cross-study causal ranking.
Early prevention during CAPOX or carboplatin–paclitaxel	Pure oral CBD 150 mg twice daily for 8 days from one day before chemotherapy; prospective non-randomized open-label cohort described in the article as CINCAN-2; public registry identity unresolved; historical CINCAN-1 controls; 54 CBD-treated (25 Carbo-Tax/29 CAPOX) [62]	Adjusted day-4 multi-frequency vibrometry favored CBD at Dig-II 250 Hz: CAPOX −1.22 (95% CI −2.05 to −0.39) and Carbo-Tax −1.76 (−2.52 to −1.02). CAPOX cold-sensitivity items also favored CBD; Carbo-Tax patient-reported outcomes did not. Mild dizziness, nausea, hypersomnia, and stomach pain were reported.	Open-label, non-randomized historical comparison with age, comorbidity, temperature, G-CSF, and missingness differences; acute first-cycle outcomes only. It supports hypothesis generation, not causal prevention or channel engagement. Context-only; not formally appraised in S4; no cross-study causal ranking.
Treatment; painful taxane-induced neuropathy	Oral cannabis capsules targeting CBD 100 mg plus THC 5 mg up to three times daily; double-blind randomized pilot; 12 women (6/6), 8 weeks [60]	Pain and several function measures improved over time in both groups without a treatment-specific pain benefit. Active cannabis had worse neuropathy ratings (*p* = 0.035), lower functional well-being in weeks 6–8 (all *p* < 0.02), and greater sleep disturbance (all *p* < 0.05); no severe adverse events. Individual BPI pain-interference contrasts were not significant (all *p* > 0.12).	Very small exploratory pilot; dose reductions were common; no effect CIs; late daily-rating missingness; no channel-engagement, axonal-protection or chronic-prevention inference. Context-only; not formally appraised in S4; no cross-study causal ranking.

No cross-study certainty grade or ranking is assigned. CI, confidence interval; SD, standard deviation; AE, adverse event. A missing or unverified CI is not evidence of equivalence. Table S3 supplies endpoint-specific sources and denominators; Table S4 identifies appraisal scope, judgments, and source limitations.

## 5. Natural Products and a Six-Domain Reporting Template

### 5.1. What a Channel Assay Can Establish

Chemically defined natural products can serve as probes even when they have no demonstrated CIPN efficacy. The inferential question is precise: does a preparation alter a named current under specified conditions, and can that observation be connected to a chemotherapy-specific phenotype? Narirutin, for example, was tested in a spared-nerve-injury model and in channel experiments [65]. That evidence does not establish efficacy in a chemotherapy model or achievable human peripheral-nerve exposure. Such examples are retained in a short pharmacological context rather than used to enlarge the core CIPN mechanism set. The compact overview in Supplementary Table S5 identifies the experimental or clinical setting of each natural-product example without ranking readiness.

Harmaline inhibited recombinant human NaV1.7 with a reported half-maximal inhibitory concentration (IC50) of 35.5 μM [66]. The study itself notes uncertainty about attaining relevant concentrations and the compound’s central nervous system liabilities. Limited comparisons with other channel subtypes do not constitute a comprehensive selectivity or safety panel. The appropriate conclusion is that a channel interaction was measured, not that the compound is a preferred CIPN candidate. Potency values without assay conditions, species, and relevant exposure can be particularly misleading when compounds are compared across papers.

Cannabidiol provides a different example. In a human Kv7.2/Kv7.3 expression system, its voltage-activation shift had a reported half-maximal effective concentration (EC50) of 214 nM [34]. This is an assay-specific functional parameter, not a universal therapeutic concentration. Drug adsorption to experimental materials, protein binding, active metabolites, and the difference between plasma and target-tissue concentrations affect exposure interpretation. The licensed oral cannabidiol product’s pharmacokinetic information cannot automatically be transferred to a topical CIPN product or a preparation containing tetrahydrocannabinol. Table 3 keeps these links separate rather than filling them from non-equivalent sources.

### 5.2. Potency, Exposure, Selectivity, and Safety

The six-domain template addresses six linked questions: which subtype and direction were measured; what functional potency was obtained; what active exposure reaches the relevant compartment; what selectivity was established; which chemotherapy and phenotype were tested; and what safety margin or oncology interaction was evaluated. This is a way of reporting the evidence, not a validated decision rule. A blank or unsearched field cannot be scored as failure, and complete reporting cannot, itself, establish efficacy. Conversely, a clinical signal does not retroactively validate an unmeasured channel mechanism [11,12,13].

An exposure comparison is interpretable only when the active molecular species, assay conditions, and compartment are compatible. Total plasma concentration should not be divided by an in vitro IC50 and presented as target coverage without accounting for binding and distribution. If only total concentration and an uncertain binding fraction are available, the resulting estimate is explicitly a sensitivity analysis rather than measured nerve exposure. For a locally delivered product, tissue distribution and duration near the sensory ending may matter more than circulating concentration. For botanical mixtures, a concentration of an assumed marker compound is not a product-wide exposure measure unless its contribution to the effect is established.

Safety must also be separated into questions. General adverse-event frequencies, neurological tolerability, an exposure-based margin against off-target effects, and compatibility with anticancer treatment do not measure the same property. A nonsignificant difference in a small trial does not establish equivalence or exclude a clinically important harm. For concomitant prevention, an intervention could alter chemotherapy disposition or antitumor action even if it reduces a neuropathy score. For post-chemotherapy local treatment, the interaction assessment can be proportionate to systemic exposure and concomitant medication, while local injury and protective sensation remain relevant.

### 5.3. Botanical Identity and Indirect Findings

Reports of botanical products should specify the authenticated species, plant part, extraction procedure, composition or marker profile, manufacturing controls, batch information, dose, and route. These fields determine whether two reports studied the same intervention. US Food and Drug Administration (FDA) botanical guidance supports product characterization and consistency, but it does not confer efficacy or regulatory approval on a product merely because a checklist is complete [67]. Supplementary Table S6 records the identity and control questions without assigning a numerical score.

Antioxidant, anti-inflammatory, mitochondrial, and behavioral findings are not discarded as biologically unimportant; they are kept outside the core channel evidence unless the study measures the relevant channel relation. Broad natural-product summaries can otherwise give the impression that dozens of interventions support a mechanism when most only share a distal behavioral endpoint. The revised account therefore limits such discussion to examples needed to explain causal or formulation boundaries. Network-pharmacology predictions are treated as hypotheses requiring experimental tests, not as demonstrations of direct binding or channel engagement.

Botanical efficacy claims also require a verifiable link between the published report, the registered trial, and the characterized study product. An unresolved intervention or registration mismatch prevents that report from supporting efficacy or translational readiness; a plausible registry identifier must not be substituted without verification.

**Table 3:** Six-domain reporting template.

Domain	Minimum Reporting Requirement	Worked-Example Boundary
Subtype and direction	Name species, protein subtype, assay, and activation/inhibition; distinguish expression from current.	Cannabidiol changes Kv7.2/Kv7.3 activation in an expression system [34].
Functional potency	Report concentration–response parameter, exposure duration, and assay conditions.	Harmaline IC50 35.5 μM; narirutin at one concentration is not an IC50 [65,66].
Relevant exposure	Match active species, unbound concentration, compartment, route, and timing.	Oral CBD-label binding does not establish free exposure from a CIPN cream [68].
Selectivity	Show tested isoforms/off-targets and concentration margins; distinguish selectivity from one positive assay.	Human NaV1.8 selectivity cannot be assigned to a different compound [25].
CIPN context	Specify chemotherapy, prevention/treatment, phenotype, species, compartment, and causal test.	Non-CIPN nerve-injury models cannot become CIPN evidence [65].
Safety and oncology context	Separate general toxicity, off-target margin, protective sensation, and anticancer compatibility.	Capsaicin patch local harms and systemic interactions are distinct questions [31].

The template is nonordinal and unvalidated: no points, aggregate score, completeness denominator, or priority status is assigned. Defined-product examples are organized under the six fields in Supplementary Tables S5 and S7. Botanical identity requirements are in Table S6. The contextual Sun et al. 2026 publication [69] is listed in Supplementary Table S7. We verified the publication metadata and abstract-level information within the present source-audit workflow; the full article and Supplementary Materials were not independently obtained. Because its registration provenance remains unresolved, it is not used as channel-engagement or product-readiness evidence [69]. The ALC indirect-comparator PK and general safety context are documented in Supplementary Table S7, with their primary sources [70,71].

## 6. Translational Tests and Research Priorities

### 6.1. Match the Model to the Mechanistic Claim

Human-induced pluripotent stem cell-derived sensory neurons and primary human dorsal root ganglion (DRG) preparations can test species-relevant current, excitability, and injury responses, but neither automatically predicts clinical benefit. Donor variability, differentiation state, and the experimental exposure schedule must be reported. Compartmentalized axon systems can examine trafficking or distal injury; neuron–glia co-cultures address interactions absent from isolated neurons. Tumor-bearing systems answer a further question about the coexistence of neuroprotection and anticancer action. These models should be chosen for the claim rather than treated as a universal ladder that every product must pass in the same order.

A useful experiment pairs a direct functional measurement with a matched perturbation. For a putative channel modulator, this means testing the intended human subtype, quantifying the relevant current or excitability change, and using genetic or pharmacological controls with appropriate specificity. For an axon-preservation intervention, structural and functional readouts remain necessary even if pain behavior improves. Transcriptomic or proteomic changes can guide these tests but cannot substitute for them. The purpose is to connect observations within a system before joining findings across species or formulations [16,50,72].

### 6.2. Distinguish Target Engagement, Physiology, Injury, and Clinical Outcomes

Target engagement is evidence that the intervention interacts with the intended molecular target in the relevant context. Electrophysiological change is a functional response, which may be mechanistically informative without uniquely identifying a subtype. Neurofilament light chain (NfL), corneal nerve measurements, and intraepidermal nerve-fiber density concern neural injury or structure. Gait and balance concern function. A symptom score concerns the patient’s experience. These measures may complement one another, but they are not interchangeable markers of channel engagement.

An injury marker can be associated with concurrent neuropathy without predicting later neuropathy, and a prognostic marker is not necessarily predictive of treatment response. Studies of NfL across chemotherapy settings illustrate the need to define timing, drug class, and context of use [73,74]. Structural or digital endpoints likewise require reproducibility and independent validation before use as enrichment criteria or surrogates [75,76,77]. Table 4 classifies the intended interpretations and the conclusions each measurement cannot support. No numerical injury threshold is proposed here for selecting a channel-targeted treatment.

### 6.3. Trial Design Considerations from Nonbinding Draft Guidance

The US Food and Drug Administration (FDA) document on prevention and treatment of CIPN was still designated draft guidance, not for implementation, when checked on 30 September 2026 [78]. Its considerations can inform the discussion of populations, endpoints, and clinical development, but they are nonbinding and do not establish that a proposed study or product is FDA-endorsed. We use the document as a development reference, not as an FDA-endorsed framework or a clinical treatment guideline.

Prevention trials should specify chemotherapy agent and regimen, baseline neuropathy, cumulative exposure, treatment timing, and the duration required to assess persistent function. Treatment trials should define established painful CIPN separately from predominantly numb or ataxic phenotypes. Outcomes should include a prespecified clinically meaningful symptom or functional endpoint and relevant harms. Changes in chemotherapy delivery are important but influenced by tumor course and clinical decisions, so they should not be interpreted as a stand-alone measure of neuroprotection.

Responder analyses and mean differences answer different questions and should be prespecified rather than selected after a null primary result. Trials also need a clear estimand, analysis population, and approach to intercurrent events such as discontinuation, dose modification, progression, or death. Missing observations should be reported by arm and reason; multiple imputation does not by itself remove bias when its assumptions are implausible. Small mechanistic trials can establish feasibility or an exposure–response relation, but they should not be presented as confirmatory clinical efficacy studies.

### 6.4. Testable Propositions

The first proposition concerns acute oxaliplatin physiology. Serial axonal-excitability measures and cold-evoked symptoms should be collected before infusion, during the acute symptom window, and after recovery, with chronic sensory function assessed separately. A prespecified within-person model can test whether the acute physiological change is associated more closely with acute symptoms than with later sensory loss. Adequate precision around a clinically justified minimum association, not simply a nonsignificant *p*-value, is needed to reject the proposed relationship. Even a positive association would not identify a sodium-channel subtype [5,6].

The second proposition concerns persistent change after repeated taxane exposure. Matched single and repeated paclitaxel schedules should be compared in human sensory-neuron or compartmentalized-axon preparations at documented unbound concentrations. Channel localization or current, excitability, and axonal structure should be measured through a prespecified washout period. Reproducible persistence across independent donor preparations, together with a perturbation that changes the proposed mediator, would support the mechanism. An effect observed only at cytotoxic exposure, or structural injury without the predicted channel relation, would not support the narrower channel-remodeling claim [16].

The third proposition is narrowed to a proposed mechanistic validation example: isolated cannabidiol should reproduce an exposure-dependent activation-voltage shift in a human Kv7.2/Kv7.3 expression system [34]. The endpoint is the vehicle-corrected shift in half-activation voltage, expressed as a positive magnitude for the predicted hyperpolarizing direction. An independent calibration experiment would measure unbound assay-medium concentrations and fit a concentration–response curve. Its half-maximal shift defines the functional threshold, and the corresponding unbound concentration defines the exposure threshold. Both estimates and their uncertainty, together with temperature, voltage protocol, and application duration, must be fixed before validation. The published nominal EC50 of 214 nM is not assumed to be a measured unbound threshold or a therapeutic concentration.

Validation would compare vehicle and below-threshold exposure with a regimen whose lower exposure-confidence bound exceeds the upper calibration bound for the exposure threshold. The expected functional effect is at least the calibrated half-maximal voltage shift. Two independently prepared validation batches, each with a sample size justified by its precision target, would test this prediction; multiple cells within one batch do not constitute independent replication. A validated positive control must demonstrate assay sensitivity. If both batches have adequate exposure and their upper confidence bounds exclude the prespecified functional effect, the exposure–functional relationship is rejected for that preparation and assay. Unidentifiable calibration, inadequate exposure or failed controls make the test inconclusive. These are proposed design rules, not experiments or data generated for this review.

This proposition tests a channel-pharmacology link, not CIPN efficacy, patient target engagement, or clinical readiness. Transfer to chemotherapy-exposed human sensory neurons requires a separate donor-based experiment. Clinical symptom benefit and preservation of axons likewise require separately justified outcomes, effect thresholds, and protocols; a reproduced channel response does not supply them.

## 7. Limitations

This review is a selected narrative synthesis. Purposive retrieval, incomplete historical logging of named-agent searches, English-language emphasis, and the absence of independent duplicate screening create selection and confirmation risks. Updated retrieval and explicit source records improve traceability but do not retrospectively make the review systematic. We do not infer study prevalence, product-level evidence absence, or comparative certainty from the selected literature. The channel scope is deliberately restricted, and a comprehensive review of P2X receptors, acid-sensing ion channels, Piezo channels, two-pore-domain potassium channels, connexins, and pannexins would require a separate retrieval strategy.

The underlying evidence also limits what can be inferred. Human physiological observations rarely resolve molecular subtypes, experimental perturbations may lack complete selectivity, and model concentrations may not match clinical unbound exposure. Clinical studies differ in prevention or treatment intent, phenotype, formulation, comparator, follow-up, and outcome definition. Result-specific appraisal cannot remove those differences or turn an uncontrolled series into a causal estimate. Single manual assessment lacks independent repeatability checks and may have interpretative errors; prospective analysis-plan locking, historical registry versions, and some abstract-only reports remain incomplete. The six-domain template is unvalidated and nonordinal; it identifies missing links without selecting a clinical intervention. Source-access status, including newly verified author-supplied appendices and figures, and the remaining unresolved records are visible in the supplementary records.

## 8. Conclusions

Selected ion-channel mechanisms contribute to chemotherapy-associated hyperexcitability, but their support depends on the agent, time, and anatomical compartment. Human oxaliplatin recordings demonstrate an axonal membrane disturbance without resolving a sodium-channel subtype. Experimental studies provide more specific hypotheses concerning sodium-channel regulation and trafficking, TRP signaling, and potassium-current suppression. These mechanisms should be distinguished from axonal degeneration and should not be generalized across all CIPN phenotypes.

A channel-targeted symptom claim requires reproducible clinical benefit with adequate exposure and preservation of useful sensory function. An axonal-protection claim also requires structural and functional preservation, and concomitant use during chemotherapy requires appropriate assessment of oncology compatibility. Natural products and clinical drugs should be evaluated against these same claim-specific standards without assuming equivalence across formulations. The six-domain reporting template organizes these questions; it neither ranks candidates nor treats unsearched fields as evidence of absence.

## Figures and Tables

**Table 1:** Selected channel-by-chemotherapy-by-phenotype matrix.

**Panel A, mechanism observations**.				
**Channel and Source**	**Agent and Model Exposure**	**System, Time and Measurement**	**Phenotype/Perturbation**	**Boundary of Inference**
NaV, subtype unresolved [6]	Oxaliplatin 130 mg/m^2^ IV over 2 h every 3 weeks, with capecitabine	Patient median motor/sensory axons, within 3 days of an early cycle and during recovery: reduced motor superexcitability; modeled slowed NaV inactivation	Acute abnormal sensations	Human in vivo physiology and model inference, not subtype-specific current or causal blockade
NaV1.6–miR-30b–TET1 [14]	Oxaliplatin 3 mg/kg i.p., alternate days ×4	Male mouse L3–L5 DRG, day-14 molecular assays: *Scn8a*/NaV1.6 abundance ↑; current only trends ↑ versus vehicle	Mechanical/cold hypersensitivity; preventive TET1 overexpression lowers NaV1.6 current versus oxaliplatin/control vector	Upstream genetic/miRNA perturbation; not selective channel necessity or established-neuropathy reversal
NaV1.7-associated sodium response [15]	Paclitaxel: qPCR 0.1 or 1 µM; current/firing 1 µM	Human donor DRG, 24 h: *SCN9A* ↑ at both tested doses; transient sodium-current amplitude/firing ↑ at 1 µM; *SCN10A* unchanged	Cellular hyperexcitability	Human ex vivo response; total transient current is not exclusively NaV1.7
NaV1.8 trafficking [16]	Paclitaxel 25 or 125 nM	Neonatal rat DRG, 24 h: distal delivery of HaloTag-human NaV1.8 ↑; separately measured endogenous rat somatic current unchanged at 25 nM	Axonal redistribution; no pain-behavior endpoint	Different channel preparations, not paired measurements of one endogenous pool; no channel-selective behavioral rescue
TRPA1 [17]	Oxaliplatin 6 mg/kg i.p., days 0, 2, 4	Male/female mice; day-7 mechanical/mild-cold and day-8–12 noxious-cold assays; sigma-1 antagonism reduces TRPA1 surface availability/responses	Preventive S1RA; TRPA1-null comparisons	Genetic plus pharmacological preclinical evidence; recombinant human protein is not patient engagement
TRPM8, harvest-group responses [18]	Single oxaliplatin 3 mg/kg i.p.	Male mouse DRG harvested at 1 or 24 h, then cultured overnight: menthol-evoked Ca^2+^ response ↑/↓ versus respective vehicle controls	Sensory-channel functional change	Different menthol protocols (300 µM/45 s vs. 100 µM/30 s); time effect not isolated; no direct cold-current measurement
TRPM8, modality-selective modulation [19]	Single oxaliplatin 6 mg/kg i.p.	Male CD1 mice, day 10: menthol-responsive DRG fraction/response ↑; recombinant-channel ligand activation inhibited by DeC-1.2 while cold activation is spared	Local DeC-1.2 3.5 µg decreases cold but not mechanical allodynia	Channel pharmacology plus phenotype-specific animal benefit; not universal TRPM8 upregulation
TRPV1 ↑/TRPM8 ↓ [20]	Paclitaxel i.p., days 1, 3, 5, 7; cumulative 8 mg/kg	Male-rat L4–L5 DRG, day 14: opposite changes in channel abundance and agonist-evoked Ca^2+^ responses	TRPV1 antagonism or TRPM8 agonism attenuates mechanical hypersensitivity	Pharmacological support; no genetic specificity or patient validation
TRPV1–18:1 lysophospholipids [21]	Paclitaxel 80 mg/m^2^ IV weekly; PrevTel-derived human cohort *n* = 35; parent intervention not resolved	Patient plasma lipidomics 24 h after dose; primary sensory neurons and hTRPV1 assays; LPC/LPG 18:1 activated TRPV1	Acute nociceptor activation; AMG9810/Trpv1-null/mutant perturbations	Human-linked mechanistic association; no patient target engagement/mediation or efficacy; cryotherapy and concomitant/prior cancer-therapy confounding
CaV3.2/T-type current [22]	Rats: paclitaxel 2 mg/kg i.p., days 0, 2, 4, 6; human cells: 1 µM	Rat DRG T-current ↑ on day 7; human donor DRG inward current/firing during acute 2–5 min exposure is inhibited by ML218 pretreatment	Early ML218 attenuates development; tested day-14 administration does not reverse established behavior	Human ex vivo plus animal pharmacology; ML218 is not CaV3.2-exclusive
KCNQ2/3 (Kv7) inhibition [23]	Acute paclitaxel 3 µM; in vivo 2 mg/kg i.p., days 1, 3, 5, 7	Rat DRG/recombinant channels: XE991-sensitive current ↓; 20 s exposure in the PIP2/current experiment	Sensory-neuron *Kcnq2* deletion attenuates mouse neuropathy phenotype	Knockout baseline fiber density is elevated; compensatory differences limit extrapolation to therapeutic opening
KCNQ/Kv7 activation [24]	Paclitaxel 2 mg/kg i.p., days 1, 3, 5, 7	Male rats; retigabine 10 mg/kg i.p. twice daily on days 1–10; tested KCNQ abundance is preserved	Preventive treatment attenuates behavior and nerve degeneration; follow-up through day 35	Preventive pharmacology despite preserved abundance; no patient efficacy or oncology safety established
**Panel B, selected modulators and the level of human evidence.**				
**Channel Relation**	**Modulator/Product**	**Evidence Type**	**Translational Status and Limit**	
NaV1.8	Suzetrigine [25,26]	Human-channel and donor-neuron pharmacology; clinical acute-pain precedent.	Approved for adult moderate-to-severe acute pain, not CIPN. No CIPN engagement or efficacy is established by these sources.	
CaV-associated α2δ subunit	Gabapentin; pregabalin [27,28]	Human CIPN randomized crossover studies; distinct populations and primary questions.	Gabapentin showed no primary symptomatic benefit; pregabalin’s primary prediction hypothesis was unsupported, with no significant secondary average-pain difference. No patient subtype engagement was measured.	
T-type calcium current	ML218 [22]	Paclitaxel rat current/behavior experiments and human donor DRG physiology.	Human ex vivo function is not patient engagement. The intervention does not exclusively identify CaV3.2, and tested late treatment did not reverse established rat behavior.	
TRPV1	Capsaicin 8% patch [29,30,31]	Defined local formulation; prospective non-interventional CIPN observation.	Human symptom observations without randomized causal inference or direct subtype-engagement measurement; no axonal-protection claim.	
TRPM8	Menthol [20,32]	Channel pharmacology and paclitaxel rat perturbation; context-dependent sensory effects.	The cited experiments do not establish clinical CIPN efficacy or engagement. They cannot be transferred to oxaliplatin cold hypersensitivity.	
Kv7/KCNQ	Retigabine; flupirtine [24,33]	Rat paclitaxel prevention experiment; oxaliplatin-exposed isolated rat and human nerve recordings, respectively.	Human isolated-nerve physiology is not in vivo patient engagement. No established-human-CIPN treatment benefit follows from these experiments.	
Kv7.2/Kv7.3	Isolated cannabidiol [34]	Human-channel expression assay without chemotherapy.	A pharmacological example, not a CIPN mechanism test. Trials of other cannabidiol formulations do not establish this target link.	

↑ indicates an increase; ↓ indicates a decrease. Directions refer only to the named measurement, not an entire channel family. Human donor cells or isolated nerves are not equivalent to target engagement in patients. Source-specific verification limits and assay schedules are given in Supplementary Table S2. Statistical significance is distinguished from a directionally higher measurement. This is a selected narrative map, not a census or ranking. DRG, dorsal root ganglion. A shared summary replaces repetitive subsection tables. NaV, voltage-gated sodium channel; CaV, voltage-gated calcium channel; TRP, transient receptor potential; Kv7, voltage-gated potassium channel family; CIPN, chemotherapy-induced peripheral neuropathy. This panel identifies selected examples, not treatment recommendations or a product ranking.

**Table 4:** Endpoint classes and limits of interpretation.

Measurement Class	What It Can Evaluate	What It Cannot Establish Alone
Target engagement	Direct binding/occupancy or a sufficiently specific channel-directed functional assay in the relevant system.	A symptom change or nonspecific electrophysiological response alone.
Excitability/physiology	Threshold tracking, current and firing measurements, with assay and compartment specified.	A molecular subtype when the assay is not subtype-resolved.
Injury/structure	NfL; corneal nerve measurements; intraepidermal nerve-fiber density [73,74,75].	Channel occupancy, analgesic response, or a validated surrogate endpoint.
Prognostic or predictive marker	A temporally defined association with later outcome, or a treatment-by-marker interaction.	Prediction from a concurrent correlation or from one favorable subgroup.
Clinical symptoms/function	Pain responders, pain interference and patient-reported sensory symptoms [63]; gait and balance measures [76].	Axonal protection from analgesia alone, or direct engagement from gait improvement.

## Data Availability

No new experimental data were generated. The extracted records, source-access notes and appraisal tables supporting this review are provided in the Supplementary Materials and Data S1.

## Supplementary Materials

The following supporting information can be downloaded at https://www.mdpi.com/article/10.3390/biom16101502/s1, Supplementary Materials: Table S1. Dated targeted-update methods and decisions; Table S2. (Record-based) study-level mechanism records and scope boundaries; Table S3. (Record-based) clinical endpoint and source-access records; Table S4. Outcome-specific appraisal worksheets and verification status; Table S5. Six-domain worked examples; Table S6. Botanical identity reporting template; Table S7. Targeted pharmacokinetic, exposure, selectivity, and safety audit. Data S1 contains machine-readable records and the citation crosswalk. Supplementary-only sources are included in the shared reference list; References [60,61,62,63,64,65,66,67,68,69,70,71,72,73,74,75,76,77,78] include both supplementary-only records and selected main-text contextual sources.

## Abbreviations

Key abbreviations used in the manuscript are defined in the table below.

ASCO	American Society of Clinical Oncology
CaV	Voltage-gated calcium channel
CBD	Cannabidiol
CI	Confidence interval
CIPN	Chemotherapy-induced peripheral neuropathy
DRG	Dorsal root ganglion
FDA	US Food and Drug Administration
Kv7/KCNQ	Voltage-gated potassium channel family/encoding genes
MIABE	Minimum Information About a Bioactive Entity
NaV	Voltage-gated sodium channel
NfL	Neurofilament light chain
SANRA	Scale for the Assessment of Narrative Review Articles
SARM1	Sterile alpha and TIR motif containing 1
THC	Tetrahydrocannabinol
TRP	Transient receptor potential
