# Supplementary material for: Selected Ion Channels in Chemotherapy-Induced Peripheral Neuropathy: Mechanisms and Translational Limits
Source: Biomolecules. 2026 Oct 15;16(10):1502. doi: 10.3390/biom16101502 (PMC13702435; doi:10.3390/biom16101502)
Supplement: Supplementary file 1 [file biomolecules-16-01502-s001.zip › Supplementary Files/biomolecules-4564419_supplement_proofed_zhangxunlang.zip!/biomolecules-4564419_supplement_proofed_zhangxunlang.pdf]

biomolecules

MDPI

Supplementary material for biomolecules 4564419

Supplementary records — literature-search cutoff: 30 September 2026; source-audit date: 4 October 2026; final proof package: 15 October 2026. Study selection is purposive. The extraction and evaluation of information sources are both done manually; no claim is made for independent and repeated manual evaluations.Sources are numbered as in the main manuscript. Historical submitted-reference identifiers are retained only in Data S1 provenance records.

Table S1 (record-based) Targeted update and selection records

This targeted update is narrative, not systematic. Query dates, access failures and the screened titles/abstracts are retained in Data S1. Publication date and indexing date are not interchangeable; older electronic publications appearing in a later issue are not counted as newly published studies. Raw counts are audit properties, not coverage or confidence measures.

Record: accessed 30 September 2026.

(TITLE_ABS:"chemotherapy-induced peripheral neuropathy" OR TITLE_ABS:"chemotherapy induced peripheral neuropathy" OR ((TITLE_ABS:paclitaxel OR TITLE_ABS:oxaliplatin OR TITLE_ABS:vincristine OR TITLE_ABS:bortezomib OR TITLE_ABS:cisplatin) AND (TITLE_ABS:neuropathy OR TITLE_ABS:neurotoxicity))) AND (TITLE_ABS:"ion channel" OR TITLE_ABS:"ion channels" OR TITLE_ABS:"sodium channel" OR TITLE_ABS:"potassium channel" OR TITLE_ABS:"calcium channel" OR TITLE_ABS:TRP OR TITLE_ABS:TRPA1 OR TITLE_ABS:TRPM8 OR TITLE_ABS:TRPV1 OR TITLE_ABS:TRPV4 OR TITLE_ABS:Nav1.7 OR TITLE_ABS:Nav1.8 OR TITLE_ABS:Nav1.9 OR TITLE_ABS:Kv7 OR TITLE_ABS:KCNQ OR TITLE_ABS:HCN OR TITLE_ABS:Piezo OR TITLE_ABS:ASIC OR TITLE_ABS:Cav3.2 OR TITLE_ABS:excitability) AND FIRST_PDATE:[2026-08-09 TO 2026-09-30]

(TITLE_ABS:"chemotherapy-induced peripheral neuropathy" OR TITLE_ABS:"chemotherapy induced peripheral neuropathy" OR ((TITLE_ABS:paclitaxel OR TITLE_ABS:oxaliplatin OR TITLE_ABS:vincristine OR TITLE_ABS:bortezomib OR TITLE_ABS:cisplatin) AND (TITLE_ABS:neuropathy OR TITLE_ABS:neurotoxicity))) AND (TITLE_ABS:"ion channel" OR TITLE_ABS:"ion channels" OR TITLE_ABS:"sodium channel" OR TITLE_ABS:"potassium channel" OR TITLE_ABS:"calcium channel" OR TITLE_ABS:TRP OR TITLE_ABS:TRPA1 OR TITLE_ABS:TRPM8 OR TITLE_ABS:TRPV1 OR TITLE_ABS:TRPV4 OR TITLE_ABS:Nav1.7 OR TITLE_ABS:Nav1.8 OR TITLE_ABS:Nav1.9 OR TITLE_ABS:Kv7 OR TITLE_ABS:KCNQ OR TITLE_ABS:HCN OR TITLE_ABS:Piezo OR TITLE_ABS:ASIC OR TITLE_ABS:Cav3.2 OR TITLE_ABS:excitability) AND FIRST_IDATE:[2026-08-09 TO 2026-09-30]

Record: accessed 30 September 2026.

("chemotherapy-induced peripheral neuropathy"[Title/Abstract] OR "chemotherapy induced peripheral neuropathy"[Title/Abstract] OR ((paclitaxel[Title/Abstract] OR oxaliplatin[Title/Abstract] OR vincristine[Title/Abstract] OR bortezomib[Title/Abstract] OR cisplatin[Title/Abstract]) AND (neuropathy[Title/Abstract] OR neurotoxicity[Title/Abstract]))) AND ("ion channel"[Title/Abstract] OR "ion channels"[Title/Abstract] OR "sodium channel"[Title/Abstract] OR "potassium channel"[Title/Abstract] OR "calcium channel"[Title/Abstract] OR TRP[Title/Abstract] OR TRPA1[Title/Abstract] OR TRPM8[Title/Abstract] OR TRPV1[Title/Abstract] OR TRPV4[Title/Abstract] OR Nav1.7[Title/Abstract] OR Nav1.8[Title/Abstract] OR Nav1.9[Title/Abstract] OR Kv7[Title/Abstract] OR KCNQ[Title/Abstract] OR HCN[Title/Abstract] OR Piezo[Title/Abstract] OR ASIC[Title/Abstract] OR Cav3.2[Title/Abstract] OR excitability[Title/Abstract]) AND ("2026/08/09"[Date - Publication] : "2026/09/30"[Date - Publication])

Newly located abstracts were not treated as full-text-verified mechanistic or efficacy results. The Wedel et al. PNAS lysophospholipid–TRPV1 record was subsequently verified from the supplied article and SAPP and is described in the 3–4 October 2026 full-text addendum as human-linked, channel-resolved mechanistic evidence; it is not used as clinical efficacy or patient target-engagement evidence.

Additional source-specific checks on 30 September 2026 addressed mechanism fields, clinical analysis populations, and source identity. Data S1 contains the current corrected mechanism records, translational product examples, item-level clinical assessment, and source-specific verification locators. Historical source-closure folders and raw retrieval artifacts are excluded from the reader-facing Data S1 archive. These checks are not an independent systematic search or duplicate human assessment.

Table S2 (record-based) Selected study-level mechanisms and scope boundaries

Every record retains chemotherapy, species, compartment, time, assay, direction, perturbation and access status. "Not extracted" does not mean unmeasured in the original study. Inspection of relevant full-text sections does not mean that all supplementary material was audited. The animal records below are source-linked reporting-completeness/availability records; no formal animal risk-of-bias rating (for example, SYRCLE) is claimed.

PARK2009 — NaV (subtype unresolved)

Park SB et al., 2009; DOI 10.1093/brain/awp219. Role: contextual_access_limited; access: FULL_TEXT_RETRIEVED_RELEVANT_SECTIONS_VERIFIED. Shared reference: [5].

System and exposure: chemotherapy: Oxaliplatin; species: Human patients; compartment: Peripheral sensory and motor axons; time: pre-infusion and within 48 h after infusion across 7–12 cycles (4–6 months), with longitudinal assessment; exposure: FOLFOX4 (85 mg/m² q2w), FOLFOX6 (100 mg/m² q2w) or XELOX (130 mg/m² q3w plus capecitabine); cumulative exposure reported in the article.

Measurement and direction: 905 axonal-excitability assessments in 58 patients using threshold tracking, recovery-cycle and threshold-electrotonus measures; acute sensory recovery-cycle changes and later cumulative sensory abnormalities (reduced refractoriness, increased superexcitability and altered 90–100-ms threshold electrotonus) preceded sensory response-amplitude loss; early abnormalities predicted final clinical outcome in 80% of the analyzed subgroup.

Phenotype and perturbation: phenotype: Acute excitability change; subsequent sensory neuropathy; causal perturbation: None; observational physiology

Human inference and limitations: human direct target action: No; threshold tracking is an indirect in-vivo excitability measure and does not identify a NaV isoform or prove causality. The study is observational and its complete dated SAP, registry history and analysis-label reconciliation were not independently obtained.

Source location: PubMed landing page: https://pubmed.ncbi.nlm.nih.gov/19745023/.

HEIDE2018 — NaV inactivation (subtype unresolved)

Heide R et al., 2018; DOI 10.1016/j.clinph.2017.11.015. Role: core; access: ACCEPTED_MANUSCRIPT_RELEVANT_SECTIONS_VERIFIED. Shared reference: [6].

System and exposure: chemotherapy: Oxaliplatin; species: Human patients; compartment: Median-nerve motor and sensory axons in patients receiving oxaliplatin/capecitabine; time: First examination within 3 days after one of the first three cycles; recovery examination during the final week before the next cycle, usually approximately 2 weeks later; one surgery-related interval was 53 days; exposure: Oxaliplatin 130 mg/m² intravenously over 2 hours every 3 weeks, with oral capecitabine 1000 mg/m² twice daily for 14 days (XELOX)

Measurement and direction: measurement: Threshold-tracking axonal excitability, quantitative sensory testing, nerve conduction and model fitting; 12 patients, 16 controls and 11 paired recovery examinations; direction: Reduced motor superexcitability correlated with abnormal-sensation intensity; model compatible with slowed NaV inactivation

Phenotype and perturbation: phenotype: Acute abnormal sensations; causal perturbation: None; observational and computational

Human inference and limitations: human direct target action: No; model inference rather than direct NaV current; limitation: Observational membrane-level inference; no measured NaV isoform-specific current, occupancy or channel-selective patient intervention

Source location: source url: https://discovery.ucl.ac.uk/id/eprint/10040295; source location: Manuscript Methods 2.1–2.2 (PDF pp. 5–6), nerve-excitability Methods 2.5.2, modeling Methods 2.5.3 and Results 3.4

ZHAO2025 — NaV1.6

Zhao S et al., 2025; DOI 10.1016/j.jbc.2025.108228. Role: core; access: FULL_TEXT_RETRIEVED_RELEVANT_SECTIONS_VERIFIED. Shared reference: [14].

System and exposure: chemotherapy: Oxaliplatin; species: Male C57BL6/J mice (25–30 g), with Tet1-floxed mice used for DRG gene manipulation; compartment: L3–L5 DRG; channel recordings from dissociated small- and medium-sized neurons; time: Behavior on days −1, 3, 7, 14 and 21; molecular comparisons include day 14. TET1 lentiviral overexpression was initiated 14 days before oxaliplatin; exposure: Oxaliplatin 3 mg/kg intraperitoneally every other day for four administrations

Measurement and direction: measurement: *Scn8a* transcript and NaV1.6 protein; zandatrigine-sensitive sodium-current component at −20 mV; neuronal firing and mechanical/cold behavior; direction: TET1/miR-30b decrease and *Scn8a*/NaV1.6 abundance increase. NaV1.6 current in oxaliplatin plus control-vector neurons shows an upward trend versus vehicle, not a demonstrated significant increase; TET1 overexpression reduces that current versus the oxaliplatin/control-vector group (reported $p = 0.0287$)

Phenotype and perturbation: phenotype: Mechanical and cold hypersensitivity; causal perturbation: DRG TET1 knockdown/overexpression and miR-30b agomir/antagomir; zandatrigine (100 nM) used for channel-current subtraction, not verified channel-selective behavioral rescue

Human inference and limitations: human direct target action: No human experiments; limitation: Do not state that chemotherapy significantly increased NaV1.6 current. Upstream pathway manipulation and preventive overexpression do not prove selective NaV1.6 necessity or reversal of established neuropathy

Source location: source url: https://pmc.ncbi.nlm.nih.gov/articles/PMC11894311/; source location: Experimental procedures: Animals, Drug administration and Whole-cell patch clamp recording; Results and Figure 6K–L, TET1 downregulates Nav1.6 protein expression and decreases Nav1.6 current

CHANG2018 — NaV1.7 expression; total transient NaV current

Chang W et al., 2018; DOI 10.1007/s12264-017-0132-3. Role: core; access: FULL_TEXT_SECTIONS_VERIFIED. Shared reference: [15].

System and exposure: chemotherapy: Paclitaxel; species: Human donor-derived primary DRG neurons; compartment: Cultured DRG neuron soma; time: 24-hour exposure; exposure: qPCR at 0.1 and 1 μM; sodium-current and firing measurements at 1 μM

Measurement and direction: measurement: *SCN9A*/*SCN10A* mRNA; whole-cell sodium-current amplitude and firing frequency; direction: *SCN9A* mRNA increases at both qPCR doses; transient current/firing increases at 1 μM; *SCN10A* mRNA unchanged

Phenotype and perturbation: phenotype: Cellular hyperexcitability; no patient pain endpoint; causal perturbation: Drug exposure; measured response is not isolated NaV1.7 current

Human inference and limitations: human direct target action: Human-cell NaV response measured; NaV1.7-specific mediation and in vivo engagement not established; limitation: Total transient current must not be relabeled pure NaV1.7 current

Source location: source url: https://pmc.ncbi.nlm.nih.gov/articles/PMC5648619/; source location: Results and Figures 4–6: concentration-specific human DRG transcript and current/firing assays

WANG2020 — NaV1.7

Wang GJ et al., 2020; DOI 10.2174/1567202617666200514113441. Role: contextual_access_limited; access: FULL_TEXT_RETRIEVED_RELEVANT_SECTIONS_VERIFIED. Shared reference: [42].

System and exposure: chemotherapy: Paclitaxel; species: male Sprague–Dawley rat (8–12 weeks); compartment: bilateral L4–L6 DRG; time: NaV1.7 mRNA/protein and pERK measured on days 7, 14 and 21; exposure: paclitaxel 2 mg/kg intraperitoneally on days 0, 2, 4 and 6 (four alternate-day doses)

Measurement and direction: measurement: NaV1.7 mRNA/protein, ERK phosphorylation, pain behavior; direction: NaV1.7 abundance and ERK phosphorylation increase

Phenotype and perturbation: phenotype: Paclitaxel-associated hyperalgesia; causal perturbation: Intrathecal MEK inhibitor U0126 attenuates abundance change and hyperalgesia

Human inference and limitations: human direct target action: No human experiments; limitation: no direct NaV1.7 current or subtype-selective perturbation was measured; intrathecal U0126 is an upstream MEK inhibitor and does not establish NaV1.7-specific causality; no human target-engagement, PK/exposure or clinical-efficacy evidence

Source location: source url: https://pubmed.ncbi.nlm.nih.gov/32407275/; source location: Methods §§2.1–2.8, Results §§3.1–3.5 and Figures 1–4

BAKER2023 — NaV1.8

Baker CA et al., 2023; DOI 10.3389/fnmol.2023.1130123. Role: core; access: FULL_TEXT_SECTIONS_VERIFIED. Shared reference: [16].

System and exposure: chemotherapy: Paclitaxel; species: Neonatal rat primary DRG neurons; tagged construct: human NaV1.8; endogenous current: rat NaV1.8; compartment: Distal axonal membrane/transport vesicles versus soma; time: 24-hour exposure in key trafficking/current experiments; exposure: 25 or 125 nanomolar; somatic-current comparison at 25 nanomolar

Measurement and direction: measurement: HaloTag-tagged human NaV1.8 surface labeling and vesicle tracking; endogenous rat somatic current measured separately; direction: Distal surface delivery/anterograde transport increase; somatic current density unchanged at tested condition

Phenotype and perturbation: phenotype: Axonal channel redistribution; behavior not tested; causal perturbation: Paclitaxel exposure only; no channel-specific rescue of pain

Human inference and limitations: human protein construct in rat neurons, not human tissue or patient engagement. Tagged axonal imaging and endogenous somatic currents are not paired measurements of the same channel pool or cell. Increased endogenous axonal current and channel-mediated pain rescue were not established.

Source location: source url: https://www.frontiersin.org/journals/molecular-neuroscience/articles/10.3389/fnmol.2023.1130123/full; source location: Methods, Expression constructs and DRG cultures; Results, distal surface expression, vesicular transport and endogenous current density; Figure 5

MARCOTTI2023 — TRPA1

Marcotti A et al., 2023; DOI 10.1093/brain/awac273. Role: core; access: FULL_TEXT_RETRIEVED_RELEVANT_SECTIONS_VERIFIED. Shared reference: [17].

System and exposure: chemotherapy: Oxaliplatin; species: Young adult male and female C57BL/6J mice (2–4 months) and TRPA1-null mice; recombinant human TRPA1 in heterologous cells; compartment: DRG sensory neurons and channel membrane trafficking; time: Oxaliplatin days 0, 2, 4; mechanical/mild-cold assays day 7 and noxious-cold days 8–12; exposure: Oxaliplatin 6 mg/kg intraperitoneally on days 0, 2 and 4; preventive S1RA 40 mg/kg intraperitoneally daily for 11 days, beginning 3 days before oxaliplatin

Measurement and direction: measurement: Agonist-evoked currents/Ca^{2+} responses, surface channel, interaction assays and behavior; direction: S1RA lowers TRPA1 surface availability/responses and attenuates hypersensitivity

Phenotype and perturbation: phenotype: Cold and mechanical hypersensitivity; causal perturbation: TRPA1 knockout plus sigma-1 pharmacology

Human inference and limitations: human direct target action: No human participants/tissue; recombinant human protein is not human target engagement; limitation: Sigma-1 antagonist effects are not evidence of direct ligand binding to TRPA1

Source location: source url: https://pmc.ncbi.nlm.nih.gov/articles/PMC9924907/; source location: Methods: Animals and drug administration; Results and figures on TRPA1-null mice and preventive sigma-1-receptor antagonism

RIMOLA2021 — TRPM8

Rimola V et al., 2021; DOI 10.3390/ijms22094962. Role: core; access: FULL_TEXT_RETRIEVED_RELEVANT_SECTIONS_VERIFIED. Shared reference: [18].

System and exposure: chemotherapy: Oxaliplatin; species: Male C57BL/6NRj mice (8–12 weeks); recombinant rat TRPM8 in HEK293 cells; compartment: DRG neurons isolated after in vivo chemotherapy; time: Mouse DRGs harvested 1 or 24 hours after oxaliplatin and cultured overnight before ex vivo calcium imaging; menthol stimulation: 1-hour harvest, 300 μM for 45 s; 24-hour harvest, 100 μM for 30 s; exposure: Single oxaliplatin 3 mg/kg intraperitoneally for mouse DRG assays; separate recombinant-cell experiments used 5 or 10 μM for 24 hours

Measurement and direction: measurement: Menthol-evoked ratiometric Ca2+ imaging; PLC/PIP2 manipulation; direction: The 1-hour harvest group shows an increased response and the 24-hour group a decreased response versus their respective vehicle controls under different menthol protocols. A direct TRPM8-expression result was not localized in the inspected source sections.

Phenotype and perturbation: phenotype: Harvest-group-specific sensory-channel responses; behavioral mediation not established by this assay; causal perturbation: PLC/PKC-pathway pharmacology in cellular assays

Human inference and limitations: human direct target action: No human tissue/participants; limitation: Different menthol concentrations and stimulation durations prevent isolation of a time effect. Agonist response is not direct cold-current measurement. The source narrative discusses unchanged expression, but direct TRPM8-expression data were not localized in the inspected sections and the original supplementary ZIP was not verified; no unchanged-expression result is asserted here.

Source location: source url: https://pmc.ncbi.nlm.nih.gov/articles/PMC8125753/; source location: Figure 1A–C (24-hour harvest) and 1D–F (1-hour harvest), Methods 4.2–4.4 and 4.8; Results 2.1 and Methods 4.10 inspected for expression-data provenance.

AIERKEN2021 — TRPM8

Aierken A et al., 2021; DOI 10.1002/advs.202101717. Role: core; access: FULL_TEXT_RETRIEVED_RELEVANT_SECTIONS_VERIFIED. Shared reference: [19].

System and exposure: chemotherapy: Oxaliplatin; species: Adult male CD1 mice aged 6–8 weeks; recombinant mouse TRPM8 in HEK293 cells; compartment: Dissociated DRG neurons after in vivo oxaliplatin, peripheral cold-sensing behavior and recombinant TRPM8; time: Day 10 after the single oxaliplatin injection for established cold-allodynia testing and DRG calcium imaging; local peptide benefit assessed through 3 hours; exposure: Single oxaliplatin 6 mg/kg intraperitoneally on day 0; DeC-1.2 3.5 μg in 20 μL intraplantarly

Measurement and direction: measurement: Whole-cell/single-channel activation; menthol-evoked DRG calcium-response amplitude and responding-cell proportion; acetone cold response and von Frey mechanical response; direction: Day-10 menthol-responsive DRG fraction and calcium-response amplitude increase. DeC-1.2 inhibits ligand-driven TRPM8 activation while sparing cold activation; the tested local dose reduces cold allodynia but not mechanical allodynia

Phenotype and perturbation: phenotype: Oxaliplatin cold allodynia; no mechanical-allodynia benefit at the tested local dose; causal perturbation: Channel-directed cyclic-peptide pharmacology

Human inference and limitations: human direct target action: No human tissue/participants; limitation: Modality- and phenotype-specific pharmacology; not evidence of uniform TRPM8 upregulation. Separate body-temperature testing does not establish a complete clinical safety window

Source location: source url: https://pmc.ncbi.nlm.nih.gov/articles/PMC8596132/; source location: Results 2.3 and Figures 6–7; Experimental Section, Animal Test—Animals and Animal Models of Pain

LIWW2024 — TRPV1 and TRPM8

Li WW et al., 2024; DOI 10.3390/ijms25115813. Role: core; access: FULL_TEXT_RETRIEVED_RELEVANT_SECTIONS_VERIFIED. Shared reference: [20].

System and exposure: chemotherapy: Paclitaxel; species: Male Sprague–Dawley rats (180–200 g); compartment: L4–L5 DRG neurons, with systemic/intrathecal TRPV1 antagonism and topical/intrathecal TRPM8 agonism; time: Paclitaxel on days 1, 3, 5 and 7; DRG channel expression and functional calcium readouts assessed on day 14; exposure: Paclitaxel intraperitoneally on days 1, 3, 5 and 7, cumulative dose 8 mg/kg; formulation contained Cremophor EL/ethanol

Measurement and direction: measurement: Immunoblot/immunostaining, agonist-evoked Ca2+ imaging, von Frey and brush responses; direction: TRPV1 expression/function increase; TRPM8 expression/function decrease

Phenotype and perturbation: phenotype: Mechanical hypersensitivity; causal perturbation: Capsazepine reduced mechanical hypersensitivity; 1% topical menthol was tested 10 minutes before behavioral testing on days 7, 14, 21 and 28; intrathecal WS-12 10 μg was administered on days 8, 10, 12 and 14

Human inference and limitations: human direct target action: No human experiments; limitation: TRPV1 upregulation and TRPM8 downregulation apply to this paclitaxel regimen and day-14 DRG assessment; pharmacological attenuation is not genetic target proof

Source location: source url: https://pmc.ncbi.nlm.nih.gov/articles/PMC11171746/; source location: Methods 4.1, 4.2 and 4.7; Results 2.2, 2.4 and 2.5

LIY2017 — CaV3.2/T-type current

Li Y et al., 2017; DOI 10.1097/j.pain.0000000000000774. Role: core; access: FULL_TEXT_SECTIONS_VERIFIED. Shared reference: [22].

System and exposure: chemotherapy: Paclitaxel; species: Rat and human donor-derived DRG neurons; compartment: DRG neuron soma; time: Rat current at day 7, expression followed through day 21; human acute exposure 2–5 minutes; day-14 reversal test negative; exposure: Rat 2 mg/kg intraperitoneally on days 0, 2, 4, 6; human cells 1 micromolar

Measurement and direction: measurement: Rat: isolated T-type current amplitude/density, CaV3.2 staining, firing and mechanical withdrawal; human: ML218-sensitive paclitaxel-evoked inward current and firing; direction: Rat: T-type current/CaV3.2 expression and firing increase; human: paclitaxel induces inward current and action-potential discharge, inhibited by ML218 pretreatment

Phenotype and perturbation: phenotype: Mechanical hypersensitivity; human-cell hyperexcitability; causal perturbation: T-type blocker ML218; early dosing attenuates development, tested day-14 administration does not reverse established behavior

Human inference and limitations: human direct target action: Human-cell electrophysiological drug response measured; no in vivo patient target engagement; limitation: ML218 is not exclusively CaV3.2-specific; follow body text for administration route

Source location: source url: https://pmc.ncbi.nlm.nih.gov/articles/PMC5303135/; source location: Methods/Results: rat dosing, ML218 prevention/reversal and human DRG electrophysiology

LIGZ2023 — CaV3.2–CaMKII–Cx43 pathway

Li GZ et al., 2023; DOI 10.1007/s10565-021-09631-y. Role: indirect/contextual pathway signal; access: FULL_TEXT_RETRIEVED_RELEVANT_SECTIONS_VERIFIED. Shared reference: [41].

System and exposure: vincristine; adult male ICR mice (20–25 g; 260 total) and primary neonatal mouse cortical astrocytes (<48 h; GFAP 85–95%); spinal dorsal-horn and primary cortical astrocyte compartments; vincristine 0.1 mg/kg intraperitoneally daily for 5 days, behavior days 0–21, spinal markers days 3/7/14/21, intrathecal treatment days 6–7 with behavior at 2 h; in vitro vincristine 3 nM for 24 h; KN-93 70 nM/10 µL, L-ascorbate 30/100/300 µM/10 µL, Gap27 14.4/43.2/144 nM/10 µL and Cx43 siRNA 50 nM/10 µL in vivo.

Measurement and direction: GFAP, p-CaMKII, CaV3.2/*CACNA1H* and Cx43 immunoblot/qPCR or immunofluorescence; Fluo-4 [Ca2+]i, qPCR/ELISA cytokines, CCK-8, JC-1, ROS, Annexin V/PI and TUNEL; vincristine increased pathway markers, calcium dysregulation, astrocyte activation, inflammatory readouts and pain behavior, while pharmacological probes and Cx43 perturbation attenuated selected responses.

Phenotype and perturbation: vincristine-associated mechanical and thermal hypersensitivity with spinal neuroinflammation; KN-93/L-ascorbate and Cx43 siRNA/Gap27 were pathway perturbations. These are pharmacological probes rather than subtype-selective CaV3.2 demonstrations.

Human inference and limitations: no human experiments; no CaV3.2 patch-clamp current/gating, *CACNA1H* genetic loss/rescue or direct target engagement; L-ascorbate, ZnCl2, KN-93 and KN-62 are not fully subtype-selective; cortical astrocytes are not spinal astrocytes; male-mouse model only; no PK/free exposure, safety or oncology-compatibility evidence.

Source location: Methods pp. 681–683; Figures 1–10 and Results pp. 683–697; Discussion pp. 696–699; DOI: https://doi.org/10.1007/s10565-021-09631-y.

WU2022 — KCNQ2/3 (Kv7)

Wu Z et al., 2022; DOI 10.3390/cells11244067. Role: core; access: FULL_TEXT_RETRIEVED_RELEVANT_SECTIONS_VERIFIED. Shared reference: [23].

System and exposure: chemotherapy: Paclitaxel; species: Adult male Sprague–Dawley rats (200–300 g); male and female Advillin-Cre sensory-neuron *Kcnq2* conditional-knockout mice; recombinant channels in CHO cells; compartment: L4–L5

DRG neuronal membrane, recombinant KCNQ2/3 channels and hindpaw intraepidermal fibers; time: Acute 3 μM paclitaxel exposure lasted 20 seconds in the PIP2/current experiment; repeated in vivo dosing on days 1, 3, 5 and 7; intraepidermal fibers assessed 4–5 weeks after treatment; exposure: 3 micromolar in key current assay; repeated in vivo 2 mg/kg intraperitoneally

Measurement and direction: measurement: XE991-sensitive M-current, recombinant KCNQ2/3 currents, withdrawal behavior and epidermal fibers; direction: Paclitaxel inhibits KCNQ current; *Kcnq2* deletion attenuates paclitaxel neuropathy phenotype

Phenotype and perturbation: phenotype: Mechanical hypersensitivity and fiber loss; causal perturbation: Channel pharmacology and sensory-neuron *Kcnq2* conditional deletion

Human inference and limitations: human direct target action: No human experiments; limitation: Conditional-knockout mice had higher baseline intraepidermal-fiber density; developmental/compensatory differences limit therapeutic extrapolation. Deletion is not equivalent to pharmacological opening. XE991 selectivity is imperfect

Source location: source url: https://pmc.ncbi.nlm.nih.gov/articles/PMC9776748/; source location: Methods 2.1–2.4; Results 3.1, 3.3 and 3.5; Discussion of conditional-knockout compensation

LIKCNQ2019 — KCNQ/Kv7

Li L et al., 2019; DOI 10.1016/j.jpain.2018.11.001. Role: core; access: FULL_TEXT_RETRIEVED_RELEVANT_SECTIONS_VERIFIED. Shared reference: [24].

System and exposure: chemotherapy: Paclitaxel; species: Adult male Sprague–Dawley rats (200–300 g); compartment: Primary sensory neurons, peripheral axons and intraepidermal nerve fibers; time: Paclitaxel on days 1, 3, 5 and 7; retigabine twice daily on days 1–10, with morning retigabine 30 minutes before paclitaxel on dosing days. Behavioral follow-up on days 12, 28 and 35; intraepidermal fibers assessed at 4 weeks; exposure: Paclitaxel 2 mg/kg intraperitoneally per administration; preventive retigabine 10 mg/kg intraperitoneally twice daily

Measurement and direction: measurement: Pain behavior, nerve morphology, *Kcnq2*/*Kcnq3*/*Kcnq5* mRNA and KCNQ5 protein; direction: Retigabine attenuates neuropathy development; tested KCNQ transcript/protein levels do not decrease

Phenotype and perturbation: phenotype: Pain-related behavior and peripheral nerve degeneration; causal perturbation: KCNQ opener retigabine

Human inference and limitations: human direct target action: No human experiments; limitation: Preventive pharmacology despite preserved measured abundance, not established clinical efficacy or neuropathy reversal. An SKBR3 cancer-cell viability assay does not establish patient-level oncology compatibility

Source location: source url: https://pmc.ncbi.nlm.nih.gov/articles/PMC7337983/; source location: Methods 2.1; Results 3.2–3.3 and associated figures

SITTL2010 — Kv7-related potassium conductance

Sittl R et al., 2010; DOI 10.1016/j.neuro.2010.07.006. Role: contextual_access_limited; access: FULL_TEXT_RETRIEVED_RELEVANT_SECTIONS_VERIFIED. Shared reference: [33].

System and exposure: chemotherapy: Oxaliplatin; species: adult male Wistar rat sural-nerve segments and four fascicles from two human sural-nerve donors; compartment: isolated peripheral myelinated axons; time: acute ex vivo organ-bath exposure with 60-min oxaliplatin incubation and washout; exposure: oxaliplatin 10–30 μM (30 μM standard rat protocol), flupirtine 10 μM in rat and 3–30 μM in human tissue; cooling was approximately 4 °C.

Measurement and direction: A-fibre compound action-potential duration, threshold tracking and 8/16-ms after-activity during warm and cooling conditions; oxaliplatin increased after-activity and cooling enhanced it, whereas flupirtine reduced late after-activity and excitability in rat and human ex vivo preparations; C-fibre responses were not the target of the reported effect.

Phenotype and perturbation: phenotype: Ex vivo axonal hyperexcitability; causal perturbation: Flupirtine pharmacology

Human inference and limitations: human direct target action: human-nerve functional response was observed, but there was no selective Kv7 isoform/current assay, patient target engagement or clinical efficacy endpoint. The model uses acute ex vivo bath concentrations and human tissue from only two donors.

Source location: Methods, Results and figures; PubMed landing page: https://pubmed.ncbi.nlm.nih.gov/20670646/.

DESCOEUR2011 — K2P TREK1/TRAAK with other excitability channels

Descoeur J et al., 2011; DOI 10.1002/emmm.201100134. Role: boundary; access: FULL_TEXT_RETRIEVED_RELEVANT_SECTIONS_VERIFIED. Shared reference: [43].

System and exposure: chemotherapy: Oxaliplatin; species: Male C57BL6J mice (20–25 g), including channel-knockout comparisons; compartment: Lumbar L1–L6 DRG and peripheral cold-sensitive pathway; time: Lumbar DRGs collected 90 hours after injection; calcium imaging followed 12–18 hours of culture; daily behavioral follow-up over 1 week; exposure: Single oxaliplatin intraperitoneally at 1, 3 or 6 mg/kg; DRG calcium-imaging preparation used 6 mg/kg

Measurement and direction: measurement: Channel transcripts and cold behavior in wild-type and TREK1/TRAAK double-knockout mice; direction: TREK1/TRAAK transcripts decrease; knockout cold phenotype overlaps oxaliplatin

Phenotype and perturbation: phenotype: Cold hypersensitivity; causal perturbation: TREK1/TRAAK double knockout; additional HCN pharmacology

Human inference and limitations: human direct target action: No human experiments; limitation: Several channels change; no isolated human K2P target validation

Source location: source url: https://pmc.ncbi.nlm.nih.gov/articles/PMC3377073/; source location: Methods: Treatments and Ca2+ imaging; Results: Oxaliplatin transcriptionally regulates a set of ion channels important for cold sensing

MEI2023 — P2X7

Mei C et al., 2023; DOI 10.1016/j.neuropharm.2023.109584. Role: boundary; access: FULL_TEXT_RETRIEVED_RELEVANT_SECTIONS_VERIFIED . Shared reference: [44].

System and exposure: chemotherapy: Paclitaxel; species: male CD-1 mice (female behavioral replication in supplement) and cultured L4–L6 DRG neurons; compartment: DRG/SGCs and lumbar spinal dorsal horn; time: single PTX 8 mg/kg i.p., Y3 intrathecal within 30 min, electrophysiology/tissue at 24 h and behavior days 1–2; exposure: Y3 1 or 4 μg intrathecal; Bz-ATP 100 μM for 10 s; Y3 100 μM or A438079 200 μM in voltage clamp.

Measurement and direction: P2X7 immunofluorescence, Bz-ATP-evoked inward current, Y3 suppression, IB4± rheobase/firing, CGRP/Iba1/GFAP; paclitaxel increased P2X7 expression and Bz-ATP current, while Y3 reduced expression/current and hypersensitivity.

Phenotype and perturbation: phenotype: Paclitaxel-associated mechanical/thermal hypersensitivity; causal perturbation: Y3 pharmacology plus A438079 comparator; docking did not verify direct binding or subtype selectivity.

Human inference and limitations: human direct target action: No human experiments; limitation: single acute dose/concentration, no direct binding/PK/unbound/selectivity/safety/oncology interaction, no oxaliplatin/docetaxel.

Source location: Methods §§2.1–2.10 pp3–4; Results §§3.1–3.7 pp5–10; Figures 1–7; DOI landing page: https://doi.org/10.1016/j.neuropharm.2023.109584.

FERRARI2015 — *Piezo2*

Ferrari LF et al., 2015; DOI 10.1186/s12990-015-0068-4. Role: boundary; access: FULL_TEXT_RETRIEVED_RELEVANT_SECTIONS_VERIFIED. Shared reference: [45].

System and exposure: chemotherapy: Oxaliplatin; species: Male Sprague–Dawley rats (200–250 g); compartment: Peripheral endothelial/sensory interface; time: Local *Piezo2* antisense 24 hours after oxaliplatin, mechanical testing 6 hours later; separate endothelial-disruption experiment 48 hours after oxaliplatin with testing 30 minutes after the perturbation; exposure: Single oxaliplatin 2 mg/kg intravenously

Measurement and direction: measurement: Mechanical hyperalgesia; endothelial *Piezo2* mRNA; direction: Local *Piezo2* antisense attenuates oxaliplatin hyperalgesia

Phenotype and perturbation: phenotype: Mechanical hyperalgesia; causal perturbation: Local antisense oligonucleotide; endothelial-function perturbation

Human inference and limitations: human direct target action: No human experiments; limitation: Local antisense and endothelial-function disruption do not provide endothelial-cell-specific genetic attribution; do not assign the 48-hour perturbation interval to the antisense experiment

Source location: source url: https://pmc.ncbi.nlm.nih.gov/articles/PMC4619430/; source location: Methods: Animals and Drugs; Results/Figure 3, oxaliplatin hyperalgesia and *Piezo2* antisense

MIKESELL2024 — PIEZO1

Mikesell AR et al., 2024; DOI 10.1126/scitranslmed.adn5629. Role: boundary; access: FULL_TEXT_RETRIEVED_RELEVANT_SECTIONS_VERIFIED. Shared reference: [40].

System and exposure: chemotherapy: Paclitaxel; species: mouse and healthy-donor human keratinocytes; compartment: epidermal keratinocytes; time: behavioral effects assessed from 2 days through 3 weeks, including day-10 mechanical assays and day-21 Piezo1-deletion follow-up; exposure: mice received paclitaxel 8 mg/kg intraperitoneally four times at 2-day intervals; cells were exposed to 1 μM paclitaxel for 30 min in the reported sensitization assays.

Measurement and direction: mechanical withdrawal/brush/pinprick behavior, keratinocyte calcium and mechanically activated currents, Yoda1 responses and PIEZO1-expressing HEK293T recordings; paclitaxel sensitized mouse and human keratinocytes and increased PIEZO1 current amplitude/conductance without increasing epidermal PIEZO1 expression; epidermal Piezo1 deletion partially reduced mouse hypersensitivity.

Phenotype and perturbation: phenotype: Mechanical hypersensitivity; causal perturbation: Keratinocyte optogenetic/chemogenetic inhibition and *Piezo1* deletion in mice

Human inference and limitations: human direct target action: human-cell functional sensitization was reported, but no patient target engagement or clinical cohort was studied. The record remains a non-neuronal preclinical boundary example; residual hypersensitivity after Piezo1 deletion and the lack of independently retrieved supplementary files limit mechanistic completeness.

Source location: Results, Methods and figures; PubMed landing page: https://pubmed.ncbi.nlm.nih.gov/39661703/.

LEMES2024 — Pannexin1

Lemes JBP et al., 2024; DOI 10.31083/j.jin2303064. Role: boundary; access: FULL_TEXT_SECTIONS_VERIFIED. Shared reference: [46].

System and exposure: chemotherapy: Paclitaxel; species: Mouse (chemotherapy experiments); rat inflammatory controls; compartment: Global Panx1 deletion; DRG pharmacology in non-chemotherapy controls; time: Acute single-dose outcomes at 1, 3, 5 and 7 hours in Figure 4 (Methods 2.7.1 describes a 5-hour observation window); repeated dosing days 1, 3, 5, 7; exposure: Paclitaxel 2 mg/kg intraperitoneally

Measurement and direction: measurement: Mechanical withdrawal thresholds in WT/Panx1-knockout mice; direction: Knockout blunts acute responses but does not prevent repeated-dose neuropathy persistence

Phenotype and perturbation: phenotype: Acute mechanical pain versus persistent allodynia; causal perturbation: Global Panx1 knockout

Human inference and limitations: human direct target action: No human experiments; limitation: Do not relabel inflammatory-model blocker efficacy as sustained CIPN efficacy

Source location: source url: https://www.imrpress.com/journal/JIN/23/3/10.31083/j.jin2303064; source location: Methods 2.3, 2.6–2.7, Results 3.3, Figure 4B/C and Conclusions on acute versus repeated chemotherapy Shared reference: [46].

Table S3 (record-based) Clinical endpoint and source-access records

These are selected clinical records, not an exhaustive human-intervention list. Primary, secondary and exploratory results remain distinct. NR means not reported in the source inspected; NOT_VERIFIED means not verified here. Missing CIs are not reconstructed from unpaired summaries or rounded percentages. Abstract-only reports cannot anchor efficacy conclusions.

Smith et al. 2013; CALGB/Alliance 170601

DOI 10.1001/jama.2013.2813; main reference [63]. Access: final publisher main text, PMC manuscript Figures 1 and 2, current official registry and the seven-page eAppendix inspected. The complete eAppendix methods, eTables 1–8 and eFigure were read; the dated prospective SAP and registry–article analysis-label correspondence remain unverified.

Design: Randomized double-blind placebo-controlled crossover; primary comparison first 5-week period

Population: Established painful CIPN; pain >=4/10; >=3 months after chemotherapy

Intervention: Duloxetine 30 mg/day week 1, then 60 mg/day weeks 2-5

Numbers: randomized: 231; randomized duloxetine first/placebo first: 115/116; treated: 220; treated duloxetine first/placebo first: 109/111; primary observed analysis n: 181; primary observed analysis duloxetine first/placebo first: 87/94; primary observed analysis status: verified from the original PMC figures and registry. The eAppendix reports 100 MCMC-based imputed datasets followed by regression imputation; the imputed analysis population is not separately stated and is not inferred.

Primary: outcome: BPI-SF average-pain change, 0–10; time: 5 weeks; duloxetine change: 1.06; duloxetine change 95% CI: 0.72–1.40; placebo change: 0.34; placebo change 95% CI: 0.01–0.66; between-group advantage: 0.73; 95% CI: 0.26–1.20; p: 0.003; analysis population: observed first-period primary outcomes: 181 (87 duloxetine-first; 94 placebo-first). Verified sensitivity analysis: multiple-imputation difference 0.750, p=0.0015; control-based pattern-mixture difference 0.667, p=0.0037. Imputed-value proportions were 7.1%, 10.8%, 13.7%, 14.6% and 14.6% for weeks 2–6. The eAppendix states that baseline covariates in participants with and without missing data were similar; no additional imputed sample size is reported.

Responders exploratory: at least 30 percent: percentages: 33.3; 17.0; RR: 1.96; 95% CI: 1.15; 3.35; at least 50 percent: percentages: 20.7; 8.5; RR: 2.43; 95% CI: 1.11; 5.3

Function: outcome: BPI interference sum, 0-70; between group improvement: 4.4; 95% CI: 0.93; 7.88; p: 0.013

Harms: Main article reports AE-related discontinuation 11% versus 1%. The supplied eAppendix eTable 7 reports grade 2–3 adverse events by initial-treatment arm (Group A n=96; Group B n=99), including dizziness, anorexia, pain, nausea, somnolence, fatigue and insomnia; no grade 4 or 5 events were listed. These exposure tables are not used to reconstruct discontinuation numerators or replace the main article percentages.

Cautions: 181 is the observed first-period primary outcome sample, not the randomized or treatment-starter cohort. The publication uses ANCOVA and reports separate missing-data sensitivity analyses; the registry labels its posted test Wilcoxon. These methods remain source-specific. The publisher narrative, PMC manuscript and figures contain conflicting no-data, completion and toxicity labels; these are retained rather than reconciled by subtraction. The eAppendix resolves the previously inaccessible sensitivity-analysis methods and estimates, but it does not provide a dated complete prospective SAP. Symptomatic benefit does not establish channel engagement, prevention or axonal restoration.

Appraisal: the specified result is assessed in Table S4 and Data S1.

Source locators: Smith et al., Methods (Statistical Analyses), Results (Patients, Pain, Pain Interference and Adverse Events), Figure 1 primary-analysis/withdrawal boxes and Figure 2 initial-treatment denominator row; ClinicalTrials.gov NCT00489411 primary result. Article, figure and registry discrepancies are retained above. Source URLs and retrieval history are in Data S1.

Registry source: https://clinicaltrials.gov/study/NCT00489411

Sources: https://jamanetwork.com/journals/jama/fullarticle/1674238; https://pmc.ncbi.nlm.nih.gov/articles/PMC3912515/; https://cdn.jamanetwork.com/ama/content_public/journal/jama/926712/joc130031_supp.pdf; https://cdn.ncbi.nlm.nih.gov/pmc/blobs/beb4/3912515/21c57e80f9ff/nihms527232f1.jpg; https://cdn.ncbi.nlm.nih.gov/pmc/blobs/beb4/3912515/b568d1fa4e9b/nihms527232f2.jpg; https://clinicaltrials.gov/api/v2/studies/NCT00489411

Rao et al. 2007; N00C3

DOI 10.1002/cncr.23008; main reference [27]. Access: supplied nine-page publisher article PDF, article body, Tables 1–3, Figure 2 CONSORT diagram and current registry record inspected. Figure 2 is now visually verified; first-period withdrawal reasons are not itemized in the figure and are not inferred.

Design: Randomized double-blind placebo-controlled crossover; two 6-week periods, 2-week washout

Population: Symptomatic CIPN >1 month; NRS pain >=4 OR ENS >=1; therefore not uniformly pain-enriched

Intervention: Gabapentin titrated to 2700 mg/day

Numbers: randomized: 115; sequences: 57; 58; week 6 observed: 38; 39; week 14 observed: 32; 36

Primary: average numerical rating scale (NRS) pain and Eastern Cooperative Oncology Group neuropathy scale (ENS), first 6-week period. Table 2 reports week-6 NRS means of 3.3 with gabapentin and 3.0 with placebo (n=38/39; P=.8); the Results text instead reports placebo 3.1. ENS means were 1.7 and 1.8 (P=.3). No between-group effect CI was reported in the inspected source. The conflicting placebo values are retained without reconstructing a precise contrast. Efficacy decisions used the first period; crossover analyses were additional.

Harms: Table 3 attributes adverse events by treatment exposure (gabapentin n=91; placebo n=89), not by 57/58 randomized sequences. Dizziness grade 2/grade 3: 6/2 versus 3/1; fatigue grade 2/grade 3: 4/1 versus 5/2. Figure 2 reports 43/57 and 47/58 on therapy at the end of week 6 and 32/41 and 36/43 on therapy at the end of week 14. The figure does not itemize first-period withdrawal reasons; no reason is inferred.

Cautions: No demonstrated benefit in the studied population; not proof of equivalence. Table 2 week-6 placebo NRS is 3.0 whereas the narrative gives 3.1; avoid extracting a precise derived contrast. The supplied Figure 2 closes the previously unverified visual flow counts, but it does not resolve individual withdrawal reasons or establish a complete-ITT analysis. The current registry gives enrollment 100 and a primary timeframe up to 14 weeks, whereas the paper reports 115 randomized and a first-period efficacy decision. The registry cannot replace the paper's observed counts or establish a prospectively locked first-period SAP.

Appraisal: the specified result is assessed in Table S4 and Data S1.

Source locators: design and randomization: PDF pp. 2111–2112: Study Design; Table 1 p. 2113; target and missingness: PDF pp. 2112–2113: Statistical Considerations; Table 2 p. 2114; withdrawal and harms: PDF p. 2115: Results; Table 3 p. 2116; registry identity: protocolSection.identificationModule.orgStudyIdInfo.id; referencesModule.references[0]; tool choice: Official Cochrane crossover teaching PDF slide 16: first-period-only row has ticks under both variants; slide footnote refers to item 5.3

Registry source: NCT00027963; NCCTG-N00C3; registry explicitly links PMID17853395/DOI 10.1002/cncr.23008 Shared reference: [27].

Sources: https://acsjournals.onlinelibrary.wiley.com/doi/full/10.1002/cncr.23008; https://clinicaltrials.gov/study/NCT00027963; https://acsjournals.onlinelibrary.wiley.com/doi/pdf/10.1002/cncr.23008 Shared reference: [27].

Hincker et al. 2019

DOI 10.1097/j.pain.0000000000001577; main reference [28]. Access: supplied 12-page article PDF, PMC/Europe PMC full text, original Figure 1 and Tables 1/2/4, current ClinicalTrials.gov protocol/results inspected. The external supplemental link remains inaccessible, but the article and official registry/protocol records are available.

Design: Randomized double-blind placebo-controlled crossover; 28-day periods with taper/washout

Population: Painful CIPN; predominantly oxaliplatin

Intervention: Pregabalin titrated to 300 mg twice daily, renal/tolerability adjustment

Numbers: enrolled: 26; sequences pregabalin first placebo first: 12; 14; first period completed by sequence: 11; 13; second period started by sequence: 11; 13; second period completed by sequence: 11; 9; started both periods from figure and registry: 24; QST predictor population from flow text: 23; article table 2 treatment column headers: 25; 25; registry secondary absolute NRS denominators: 24; 24; selected published paired estimate n: UNRESOLVED: 24 started both periods and registry lists 24, but article Table 2 headers list 25 per treatment and Discussion states 23 for primary/secondary analyses. Do not unconditionally identify 24 as the published P=0.08 paired-analysis denominator; safety per exposure: 25

Primary: outcome: Between-treatment difference in baseline mechanical-pain-threshold versus pain-reduction slopes; average pain slope p: 0.92; worst pain slope p: 0.6

Secondary: average pain reduction points: 0.8; 0.5; SD: 1.1; 1.1; p: 0.08; 95% CI: NR; average pain percent reduction: 22.5; 10.7; percent reduction p: 0.23; source of existing 0 8 vs 0 5: Article Results: absolute average daily pain reductions 0.8 (SD 1.1) and 0.5 (SD 1.1); paired P=0.08; exact analysis denominator not consistently reported; registry absolute NRS change separately reported: means: -1.0; 0.3; SD: 1.13; 1.1; n: 24; 24; locator: resultsSection.outcomeMeasuresModule.outcomeMeasures[1]; caution: Registry uses change rather than reduction wording. Even after accounting for direction, these values do not reproduce the published 0.8/0.5 reductions. Do not combine, substitute, derive a paired CI, or call the discrepancy resolved.

Harms: withdrawal during exposure: 5; 1; dizziness: 17/25; 4/25; somnolence: 22/25; 11/25; withdrawal attribution: Five withdrawals occurred during pregabalin exposure and one during placebo, but the Discussion identifies non-pregabalin explanations for two active-period withdrawals; do not call all five drug-caused.

Cautions: Primary outcome is prediction, not an analgesic superiority endpoint; LOCF; early recruitment termination; 73% correctly guessed treatment; Oxaliplatin worst-pain percentage finding is post hoc, not confirmatory; The actual sequence counts are 12/14 (Figure 1 and Table 1), not merely inferred from blocked 1:1 randomization; Published flow supports 24 participants starting both periods, not an unqualified 24-person denominator for the selected published paired contrast; Table 2/Discussion use conflicting labels; Protocol/SAP version 25 January 2017, uploaded 6 March 2019, is available. It prespecifies secondary absolute average/maximum pain comparisons using paired t tests if normally distributed, with Wilcoxon fallback. Public upload postdates completion; it does not independently establish the exact pre-unblinding locked version or adherence; Protocol p. 13 specifies the blinding question on day 5 of period 1, before the days 24–28 diary endpoint; p. 10 instead includes generic end-of-study wording. Article Section 2.4 says during the first treatment sequence, and Results report 19/26 correct guesses. Actual day 5 administration is not independently documented, but an end-only interpretation is unsupported; Protocol p. 18 defines missing-data approaches conditional on the proportion of missing primary outcomes (<10%: per-protocol plus ITT/LOCF sensitivity; >10%: mixed-effects/FIML). Article Section 2.7 reports LOCF for missing final-week diaries and does not report implementing FIML. Primary-predictor missingness and secondary-pain diary missingness are not identical denominators; nonadherence to a specific threshold must not be asserted without the required counts; Article Table 2 reports placebo 50% average-pain responders as 3 (8%), an internal count/percentage inconsistency. Do not derive responder contrasts/NNTs from that row; Article Results reports average-pain primary slope comparison P=0.92, whereas Abstract/Discussion state 0.97. Retain source labels; neither is the secondary P=0.08 result; Placebo-end plasma contained no detectable pregabalin in 23 tested participants; this supports absence of residual circulating drug at sampling but does not prove absence of every carryover mechanism.

Appraisal: the specified result is assessed in Table S4 and Data S1, corrections_v6/current_appraisal.

Source locators: randomization and flow: Article Section 2.4; Figure 1; Table 1; registry participantFlowModule periodsI/II; target and numerical difference: Article Section 2.7; Results paragraph beginning Figure 2 and Table 2; Table 2 header/footnote; registry outcomeMeasures[1]; protocol analysis: Protocol pp. 12, 15, 17–18:Section 2.4.5.2, Section 2.4.7, Section 2.4.9; blinding timing: Protocol p. 13:Table 1 footnote and Subject Blinding Perception; p. 10:Study Design continuation; article Section 2.4 and Results blinding paragraph; protocol provenance: Registry documentSection.largeDocumentModule.largeDocs[0]:date2017-01-25, uploadDate2019-03-06T08:29

Sources: https://pmc.ncbi.nlm.nih.gov/articles/PMC6687437/; https://clinicaltrials.gov/study/NCT02394951; https://cdn.clinicaltrials.gov/large-docs/51/NCT02394951/Prot_SAP_000.pdf

Shinde et al. 2016; ACCRU pilot

DOI 10.1007/s00520-015-2807-5; supplementary scientific context. Access: supplied seven-page publisher article PDF inspected; abstract, Methods/Results, Figure 1, Figure 2 and Tables 1–4 read. Shared reference: [64].

Design: Randomized placebo-controlled prevention pilot

Population: Weekly paclitaxel 80 mg/m² for 12 weeks

Intervention: Pregabalin 75 mg twice daily

Numbers: 46 randomized (23 pregabalin/23 placebo); five cancelled, withdrew or lacked baseline data (4/1); first-cycle primary-evaluable n=41 (19 pregabalin/22 placebo).

Primary: maximum worst pain over the first six days after cycle 1; pregabalin mean 2.6 (SD 2.5) versus placebo 3.2 (SD 3.0), p=0.56; no reported effect CI.

Secondary: maximum average pain p=0.48; AUC of worst/average/least pain p=0.62/0.22/0.07; EORTC QLQ-CIPN20 sensory growth-curve/AUC p=0.88/0.46; no consistent tingling or pain benefit, with only a weak numbness signal.

Harms: no statistically significant or clinically apparent between-arm differences in the reported toxicity categories; no complete exposure-level AE table was used to reconstruct rates.

Cautions: multicentre prevention pilot; five participants were not primary-evaluable and no effect CI was reported. Acute paclitaxel pain and questionnaire outcomes do not establish chronic-CIPN prevention.

Sources: https://link.springer.com/article/10.1007/s00520-015-2807-5; https://pubmed.ncbi.nlm.nih.gov/26155765/ Shared reference: [64].

Salehifar et al. 2020

DOI 10.1007/s40261-019-00882-6; supplementary scientific context. Access: supplied nine-page publisher article PDF inspected; Methods/Results, Figure 1 and Tables 1–3 read. Shared reference: [52].

Design: Randomized double-blind phase II active-comparator trial

Population: Breast-cancer patients with established taxane sensory neuropathy (NCI-CTCAE grade >=1 and VAS >=4); 30 received paclitaxel and 52 docetaxel; chemotherapy was ongoing for 52 participants and completed for 30.

Intervention: Pregabalin 150 mg/day versus duloxetine 60 mg/day, 6 weeks

Numbers: 108 assessed; 26 excluded (11 ineligible, 15 declined); 82 randomized and analyzed (pregabalin 40, duloxetine 42); no reported loss to follow-up.

Primary/secondary results at six weeks: VAS ≥33% improvement 37/40 (92.5%) versus 16/42 (38.1%), p<0.001; NCI-CTCAE sensory improvement 37/40 (92.5%) versus 13/42 (31%), p<0.001; PNQ improvement 36/40 (90%) versus 13/42 (31%), p<0.001. Adjusted GEE estimates for duloxetine versus pregabalin were VAS +4.71 (95% CI 2.30–7.13), NCI grade +0.36 (0.21–0.51), and PNQ +0.56 (0.38–0.75), all p<0.001.

Cautions: Pregabalin was 75 mg daily for one week then 75 mg twice daily; duloxetine was 30 mg daily for one week then 30 mg twice daily. No placebo arm; single-centre breast-cancer active-comparator study. Mild adverse events were reported, with somnolence/dizziness more frequent with pregabalin and nausea/vomiting more frequent with duloxetine; no AE discontinuations were reported. Response CIs were not reported.

Sources: https://link.springer.com/article/10.1007/s40261-019-00882-6 Shared reference: [52].

Durand et al. 2012; EFFOX

DOI 10.1093/annonc/mdr045; main reference [53]. Access: supplied six-page publisher PDF inspected; article body, Figure 1 and Tables 1–4 read.

Design: Randomized double-blind placebo-controlled acute-neurotoxicity study

Population: Oxaliplatin acute neurotoxicity, median 4.5 previous cycles

Intervention: Venlafaxine 50 mg before infusion, then 37.5 mg twice daily days 2-11

Numbers: 54 assessed; 6 excluded (2 eligibility, 4 patient decision); 48 randomized (24/24); 20 venlafaxine and 22 placebo assessable for neurotoxicity. The trial was conducted in six French hospitals after a median 4.5 oxaliplatin cycles; mean cumulative dose at inclusion was 684.6 mg.

Primary: 100% acute relief under treatment, reported as 31.3% versus 5.3%, p=0.03. Secondary ≥50% relief 68.8% versus 26.3%, p=0.02; pins-and-needles p<0.001; cold-triggered pain p=0.06; functional impairment p<0.001. At three months, grade-3 toxicity was 0% versus 33.3%, p=0.03, and grade-0 toxicity 38.5% versus 5.6%, p=0.06. No effect CI was reported.

Cautions: Small trial stopped early. The published percentages and Table 4 adverse-event denominators do not map cleanly to 20/22 assessable or 24/24 randomized participants; event counts and CIs were not reconstructed. Grade 1–2

nausea, vomiting, asthenia/somnolence and orthostatic hypotension were more frequent with venlafaxine. Acute symptom relief is not chronic-CIPN prevention.

Sources: https://pubmed.ncbi.nlm.nih.gov/21427067/; https://academic.oup.com/annonc/article-abstract/23/1/200/161787

Zimmerman et al. 2016; MC11C4

DOI 10.1007/s00520-015-2876-5; main reference [54]. Access: Complete PMC author-manuscript body obtained through NCBI EFetch and PMC HTML.

Design: Randomized double-blind placebo-controlled prevention pilot

Population: Colorectal cancer receiving modified FOLFOX6

Intervention: Venlafaxine XR 37.5 mg twice daily versus placebo during oxaliplatin 85 mg/m^2 every two weeks in FOLFOX; start before cycle 1 or 2 and continue one week after last FOLFOX

Numbers: accrued: 50; randomized groups: 25; 25; treated groups: 24; 24; treated groups source: Main Results text reports two nonstarters and 48 treated; Figure 1 received-treatment boxes instead read 25/25, retained as unresolved inconsistency; primary evaluable figure 1: 43; primary evaluable groups figure 1: 22; 21; group order: venlafaxine; placebo; timepoint denominators: source: Figure 3a, cycle 1 to 12, visually inspected; venlafaxine: 20; 22; 17; 18; 16; 16; 13; 10; 5; 5; 5; 4; placebo: 21; 18; 17; 18; 18; 14; 12; 8; 11; 10; 7; 4; registry cycle1 groups: 21; 20; registry flow completed groups: 16; 18

Primary: outcome: Longitudinal EORTC QLQ-CIPN20 sensory score; p: 0.55; effect 95% CI: NR in inspected text

Secondary: motor p: 0.25; autonomic p: 0.23

Harms: NCI CTCAE v4 adverse events were assessed before each FOLFOX cycle. Grade 3 hypertension occurred in one patient in each arm; severe neutropenia was reported in five venlafaxine-treated patients and three placebo-treated patients. No severe gastrointestinal toxicities were reported in either arm. The aggregate toxicity window and safety-analysis denominator were not separately specified in this paragraph; event percentages and confidence intervals were not reconstructed. These observations during concurrent FOLFOX do not establish attribution to venlafaxine, and severe events should not be relabeled as serious adverse events. Separately, Figure 1 records one placebo participant stopping because of an unspecified serious adverse event. Its nature and attribution are not reported; it is not equated with any listed toxicity event.

Cautions: No consistent acute/chronic prevention benefit; pilot precision limited; absence of significance is not proof of equivalence; Selective early-cycle cold-symptom differences are secondary and not multiplicity-adjusted; Figure 1 primary-evaluable total 43 (22/21), varying Figure 3 cycle-specific numbers, and current registry flow-completed 16/18 must not be treated as the same denominator. Figure 1 received-treatment boxes 25/25 conflict with the main-text 24/24; Current registry labels cycle-1 sensory as primary and posts P=.87, with 21/20 participants; this differs from the published longitudinal primary P=.55 and from Figure 3a cycle-1 arm counts 20/21. No silent reconciliation or interchange of estimands is permitted; Atherton et al. (2016) [56], Table 3, reports MC11C4 booklet compliance: 498/543 expected booklets (91.7%; 47 persons). This is not arm-specific sensory-outcome completeness or informative-missingness sensitivity; its different denominators do not replace the 50-person trial cohort.

Appraisal: the specified result is assessed in Table S4 and Data S1.

Sources: https://pmc.ncbi.nlm.nih.gov/articles/PMC4939800/; https://pubmed.ncbi.nlm.nih.gov/26248652/; https://clinicaltrials.gov/study/NCT01611155; https://cdn.ncbi.nlm.nih.gov/pmc/blobs/14fa/4939800/f0907607e7f3/nihms774687f1.jpg; https://cdn.ncbi.nlm.nih.gov/pmc/blobs/14fa/4939800/1add39d6d7c7/nihms774687f3.jpg; https://pmc.ncbi.nlm.nih.gov/articles/PMC5133154/

Lavoie Smith et al. 2026; Alliance A221805

DOI 10.1200/OA-25-00107; supplementary scientific context. Access: supplied 11-page publisher PDF, complete accepted manuscript body and Tables 1–4, publisher abstract, current registry, and the official 131-page ClinicalTrials.gov protocol/SAP (Version Date 01/11/24, Update #05) inspected. The current NCT04137107 record has no posted results and is not a substitute for the published phase II result. The dated public protocol confirms the phase-II design and primary analysis specification, but the exact pre-unblinding version lock and registry-history correspondence remain unverified. Shared reference: [57].

Design: Randomized double-blind multicentre three-arm noncomparative phase II dose-screening design; placebo calibration arm. The phase II portion was explicitly not powered for a formal duloxetine-versus-placebo superiority comparison.

Intervention: Duloxetine 30 or 60 mg/day versus placebo

Numbers: randomized: 199; randomized groups 30 60 placebo abstract: 66; 66; 67; randomized groups 30 60 placebo main results and Table 1: 66; 67; 66; randomized arm count status: SOURCE_DISCREPANCY: do not silently select one without source label; mITT evaluable groups 30 60 placebo: 46; 47; 50; mITT total: 143; toxicity evaluable groups 30 60 placebo Table 4: 63; 61; 61; toxicity evaluable total: 185; withdrew before protocol therapy: 14; missing postbaseline OIPN: 42

Primary: outcome: Maintenance of no/mild sensory symptoms, not symptomatic improvement from baseline: highest of six QLQ-CIPN20 numbness/tingling/pain items <=2 and no study withdrawal because of sensory OIPN; additional definition: Participants were also responders if their highest recorded score was <=2 at death, progression, or two weeks after treatment completion; any item>2 or withdrawal for sensory OIPN was failure; time: Abstract reports assessment in weeks 19-21; main design specifies maintenance during treatment and two-week follow-up, and evaluation after standard cumulative oxaliplatin 510-520 mg/m². Six-month FOLFOX participants did not receive study medication in the final three months of oxaliplatin therapy; mITT eligibility: Eligible at registration, initiated oxaliplatin and submitted at least one postbaseline OIPN questionnaire; responders 30 60 placebo: 30; 31; 34; denominators 30 60 placebo: 46; 47; 50; percentages 30 60 placebo: 65.2; 66.0; 68.0; 90% CI percent 30mg: 52.1; 76.8; 90% CI percent 60mg: 53.0; 77.4; 90% CI percent placebo: 55.5; 78.8; CI method: Reported two-sided 90% exact Clopper-Pearson intervals, not 95% intervals; between group effect CI: NOT_REPORTED_IN_INSPECTED_SOURCE for a prespecified primary superiority contrast; no derived CI produced; interpretation: Neither duloxetine dose was more promising than placebo in this dose-screening setting; do not describe as proof of equivalence or a confirmatory superiority trial

Harms: grade ≥3 any attribution counts 30 60 placebo: 19; 22; 19; denominators 30 60 placebo Table 4: 63; 61; 61; grade 4 any attribution: 7; deaths: 2; attribution note: Among the severe events discussed in the adverse-effects paragraph, one grade 4 hypokalemia in the 60 mg arm was considered possibly study-drug-related. Do not claim all-cause severe events/deaths were duloxetine-caused or that none occurred; grade ≥1 solicited possibly related fatigue counts 30 60 placebo: 29; 34; 33; grade ≥1 solicited possibly related nausea counts 30 60 placebo: 32; 26; 28

Cautions: Abstract versus Results/Table 1 swaps 60 mg/placebo randomized counts 67/66; total 199 and mITT 46/47/50 are consistent; Main adverse-effects text prints placebo grade ≥3: 19 as 31.3%, whereas Table 4 denominator 61 would not yield that rounded percentage; report published count with separately source-labelled denominator, not a silently corrected percentage; Table 3 has inconsistent sensitivity counts (e.g., 30 mg nonresponder count 23 alongside 52.4% of 63); no sensitivity counts are promoted as independently corrected data; Table 4 placebo nausea CI upper endpoint is malformed in the displayed text (0.58.4); no guessed numerical CI is reused; Current registry phase II time frame is up to one month post-oxaliplatin, not identical to published two-week poststudy-treatment rules. Pre-unblinding version correspondence remains unverified; Retained as detailed supplementary prevention context, not a confirmatory efficacy anchor or a formally appraised result in the selected main clinical table.

Source locators: previous source locations: source url: https://pmc.ncbi.nlm.nih.gov/articles/PMC13120771/; location: Methods: Study Design, Intervention, Statistical Analysis; Results: Participants, Primary Outcome, Adverse Effects; Tables 1-4; source url: https://clinicaltrials.gov/api/v2/studies/NCT04137107; location: hasResults=false; protocolSection.outcomesModule; current record includes both phase II and phase III planned outcomes; source url: https://ascopubs.org/doi/10.1200/OA-25-00107; location: Publisher abstract Methods and Results; inspection: Indexed official publisher output were read; https://cdn.clinicaltrials.gov/large-docs/07/NCT04137107/Prot_SAP_000.pdf; location: official 131-page protocol/SAP, Version Date 01/11/24, Update #05, §§13.1, 13.5 and 12.3.2 (phase-II design, primary endpoint/90% exact CI and early-discontinuation sensitivity). The exact pre-unblinding version lock and registry-history correspondence remain unverified. Shared reference: [57].

Registry source: https://clinicaltrials.gov/study/NCT04137107

Sources: https://ascopubs.org/doi/10.1200/OA-25-00107; https://ascopubs.org/doi/pdfdirect/10.1200/OA-25-00107; https://pmc.ncbi.nlm.nih.gov/articles/PMC13120771/; https://clinicaltrials.gov/api/v2/studies/NCT04137107 Shared reference: [57].

D'Andre et al. 2024

DOI 10.1089/can.2023.0253; main reference [59]. Access: PMC main methods/results read; official 45-page posted protocol/SAP PDF retrieved. Outcome-relevant eligibility, randomization and masking sections (5; 6.1-6.4e), assessment schedule, amendment/version dates and statistical section 16 were inspected for the primary first-period assessment. A complete

page-by-page protocol audit and pre-unblinding plan lock were not established. Original PMC Figures 1 and 2 and current registry adverse-event results additionally inspected on 30 September 2026; week-2 denominator now verified.

Design: Double-blind randomized placebo-controlled crossover; two 2-week periods; no washout

Population: Established painful/tingling CIPN >=4/10, chemotherapy completed >3 months earlier

Intervention: CBD-isolate cream, 250 mg/1.7 oz, applied twice daily to affected hands and/or feet. Protocol Section 15 specifies 8 mg/day for hands only or feet only and 16 mg/day when both are treated; 0% THC is stated by reference to the product COA. This was a specially manufactured preparation without the extra herbals of the marketed cream.

Numbers: randomized: 40; randomized sequences: 20; 20; overall analysed sequences: 20; 18; primary week 2 analysis n: 35; primary week 2 analysis sequences: 19; 16; primary week 2 analysis status: VERIFIED in Figure 1 primary-endpoint boxes and Figure 2 week-2 denominator row; figure 2 week specific sensory n: time: baseline; week 1; week 2; week 3; week 4; CBD first: 20; 19; 19; 19; 19; placebo first: 17; 17; 16; 17; 15; primary missingness components: CBD first: One missing week-2 CIPN20 observation among 20 in the overall analysis; placebo first preanalysis: One ineligible and one cancelled before starting treatment among 20 randomized; placebo first primary: One missing baseline CIPN20 and one cancelled week-2 CIPN20 among 18 in the overall analysis; registry shared primary denominators: 20; 17; registry shared primary denominator note: Posted once for the weeks 1-4 series; not a week 2-specific complete-case denominator

Primary: outcome: First-period change in EORTC QLQ-CIPN20 sensory score; time: 2 weeks; finding: No first-period sensory benefit demonstrated; not proof of equivalence; mean difference 95% CI: NOT_REPORTED_IN_INSPECTED_SOURCE; arm-level registry percent-of-baseline CIs are not a between-group raw change-score CI; analysis population: Week-2 first-period complete primary sensory observations: 19 CBD-first and 16 placebo-first (35 total); available plan: Protocol Section 16.1 specifies the week-2 two-sample test and between-arm mean-change 95% CI, as well as repeated-measures mixed models. Longitudinal modelling is not itself a demonstrated analysis departure. Exact prospective version locking and full reported-output correspondence remain unverified.

Harms: paper: No skin irritation/rash/blisters/open areas reported in the first two weeks and no suicidal thoughts over four weeks. One participant had a skin reaction at week 4 while using placebo after crossover. This is not an assertion of zero all-cause adverse events; registry time frame: Up to 28 days; registry all cause nonserious affected at risk: EG000 CBD then placebo: 2; 20; EG001 placebo then CBD: 0; 20; EG002 CBD then placebo crossover: 0; 19; EG003 placebo then CBD crossover: 0; 17; registry event terms in EG000: Spinal fracture: one participant/one event; Back pain: one participant/one event; Hypotension: one participant/one event; registry serious events and deaths: Zero affected in each of the four posted groups; interpretation limit: These are registry-labelled sequence/period groups over an up-to-28-day reporting window; event dates and causal attribution to CBD are not established. Do not call the three events CBD-caused or assign exact first-period dates. Two affected people is not three affected people.

Cautions: Use 19/16 for the first-period primary sensory outcome, not 20/18 overall analysed or the registry's shared 20/17 series denominator; No between-group primary change-score estimate with confidence interval is reported in the inspected article. Registry arm-level percent-of-baseline CIs are not a between-group raw change-score CI; do not manufacture a contrast; The first-period comparison is unaffected by treatment carryover from a prior study period, but missing outcomes and reporting/scale correspondence still matter; Figure 1 crossover data boxes state 19/16, Figure 2 week 3 and 4 counts vary, and the registry crossover flow says 19/17; use endpoint/time-specific counts rather than force reconciliation; The null finding concerns this product and dose, not all cannabinoid formulations; Late upload or a post-enrollment protocol version does not by itself prove post-unblinding changes. Conversely, the available version cannot certify the original prospectively locked plan; Protocol Appendix VII states that no formal PK studies had been done for this particular product. The referenced investigator-brochure COA was not independently retrieved in this source check; The registry's all-cause adverse-event module covers up to 28 days and sequence/period-labelled groups; it is not a verified first-period CBD-causality count.

Appraisal: the specified result is assessed in Table S4 and Data S1.

Source locators: previous source locations: source url: https://cdn.ncbi.nlm.nih.gov/pmc/blobs/b404/11685298/9c721c09c51d/can.2023.0253_figure1.jpg; location: Figure 1 primary-endpoint and missingness boxes; source url: https://cdn.ncbi.nlm.nih.gov/pmc/blobs/b404/11685298/7a790ba2f63f/can.2023.0253_figure2.jpg; location: Figure 2 denominator rows at baseline and weeks 1-4; source url: https://clinicaltrials.gov/api/v2/studies/NCT05388058; location: resultsSection.adverseEventsModule; outcomeMeasuresModule primary denoms and classes; participantFlowModule; source url:

https://pmc.ncbi.nlm.nih.gov/articles/PMC11685298/; location: Results: Changes in neuropathy scores and toxicity during study periods, final paragraph; analysis plan: https://cdn.clinicaltrials.gov/large-docs/58/NCT05388058/Prot_SAP_002.pdf; Section 16.1, PDF p. 27 (printed protocol p. 26)

Registry source: https://clinicaltrials.gov/study/NCT05388058

Sources: https://pmc.ncbi.nlm.nih.gov/articles/PMC11685298/; https://cdn.clinicaltrials.gov/large-docs/58/NCT05388058/Prot_SAP_002.pdf;
https://cdn.ncbi.nlm.nih.gov/pmc/blobs/b404/11685298/9c721c09c51d/can.2023.0253_figure1.jpg;
https://cdn.ncbi.nlm.nih.gov/pmc/blobs/b404/11685298/7a790ba2f63f/can.2023.0253_figure2.jpg; https://clinicaltrials.gov/api/v2/studies/NCT05388058

Weiss et al. 2025

DOI 10.3389/fonc.2025.1590168; main reference [61]. Access: Full article, tables and complete six-page supplement.

Design: Randomized double-blind placebo-controlled trial, 12 weeks

Population: Grade 2-3 established CIPN after completed chemotherapy

Intervention: Plant-derived oral CBD 125.3-135.9 mg/day plus THC 6.0-10.8 mg/day; product also contains minor cannabinoids and terpenes, not a purified two-compound isolate

Numbers: randomized: 46; groups: 24; 22; completed primary: 43; primary groups: 23; 20; identified Figure 1: 230; ineligible Figure 1: 132; eligible Figure 1: 98; eligible narrative abstract: 124; eligible count status: SOURCE_DISCREPANCY: original flow diagram 98 conflicts with main narrative/abstract 124; do not silently reconcile; consented Figure 1: 54; withdrew before randomization Figure 1: 8; randomized Figure 1: 46; randomized active placebo: 24; 22; primary week 12 observed active placebo: 23; 20; primary week 12 missing active placebo: 1; 2; primary week 12 missing reasons: NOT_REPORTED in full paper, full six-page supplement, original flow diagram or current registry; the eight consent withdrawals precede randomization and are not reasons for these three missing outcomes; registry enrollment actual: 56; registry enrollment status: Different from published 54 consented/46 randomized; do not substitute registry enrollment for analytic n

Primary: outcome: Baseline-to-week 12 EORTC QLQ-CIPN20 sensory change; improvement active placebo: 13.5; 9.3; SD: 12.5; 10.3; p: 0.24; 95% CI: NOT_REPORTED_IN_INSPECTED_SOURCE for the primary unadjusted baseline-to-week 12 between-group change; no derived interval calculated

Exploratory: adjusted sensory coefficient: -10.4; 95% CI: -20.5; -0.3; p: 0.044; adjusted BPI average pain: -0.04; pain 95% CI: -0.8; 0.7; pain p: 0.916

Harms: No serious adverse events or AE-related withdrawals reported

Cautions: Exploratory model includes all 46 with >=8-week data, adjusted for time/baseline grade; not the primary complete-case change comparison; No multiplicity adjustment; do not privilege nominal secondary significance; Supplement sample-size calculation uses alpha 0.025 whereas article analysis section uses 0.05; clarify without assuming selective reporting; Table 1 caption and body denominators differ; abstract/table liver-laboratory counts differ; Supplement Table S1 caption says n=43 but body is all 46 (22 placebo/24 active). Active gelcaps also contain CBC, cannabidivarin, CBG and terpenes; placebo has minor terpenes. Restrict inferences to this tested preparation; The flow diagram gives 98 eligible, whereas the narrative/abstract says 124; neither count is needed for the retained primary efficacy summary; No AE-related removal was reported, but the actual reasons for three missing week 12 primary observations remain unspecified. Similar harms and at least eight-week follow-up do not establish absence of outcome-dependent missingness; Supplement Tables S4/S5 concern time since chemotherapy, not MNAR sensitivity analysis. The all-46 exploratory mixed model is not a validation of the 43-person week 12 primary result against informative missingness; The manuscript's 15% attrition statement has no explicit denominator matching the three missing primary observations among 46 randomized. Report the observed counts instead of importing 15% as endpoint-specific missingness; Current registry first posted 21 May 2020 and was last submitted for update 29 August 2022; the paper reports trial conduct ending 8 August 2022. No dated locked SAP is attached, and the current registry does not establish prospective prioritization of sensory week 12 among its seven primary outcome families.

Appraisal: the specified result is assessed in Table S4 and Data S1.

Sources: https://www.frontiersin.org/journals/oncology/articles/10.3389/fonc.2025.1590168/full;
https://pmc.ncbi.nlm.nih.gov/articles/PMC12588866/

Haney et al. 2025

DOI 10.1089/can.2025.0028; main reference [60]. Access: Nine-page publisher PDF inspected; abstract, article body, Figure 1, Figures 2–3 and reported results read. The official 25-page public protocol-summary/consent package for NCT03782402 (Protocol 7635, version 30 July 2021) was also retrieved; design, dose and statistical-analysis sections were inspected. This is not a complete dated SAP; current registry history/results were not independently audited. The planned-sample discrepancy (96 in the study-plan section versus 98 in the consent section) is retained. Protocol: https://cdn.clinicaltrials.gov/large-docs/02/NCT03782402/Prot_SAP_ICF_000.pdf (protocol/version p1; aims and groups pp3–4; mixed-effects analysis p16).

Design: double-blind randomized placebo-controlled 8-week pilot; conducted 2019–2021 and analyzed 2023–2024.

Population: 12 women with painful taxane-induced peripheral neuropathy after paclitaxel or docetaxel for breast cancer; six randomized to active cannabis and six to placebo.

Intervention: GMP-prepared oral capsules containing CBD 100 mg plus THC 5 mg per capsule, instructed up to three capsules/day (target 300 mg CBD plus 15 mg THC/day); placebo contained <0.1 mg CBD/THC.

Numbers: 12 randomized (6/6); all completed the 8-week study and weekly assessments. Mean capsules/day were 2.0 ± 0.4 active and 2.4 ± 0.4 placebo because dose reductions were requested in both groups; daily-response completion declined during weeks 7–8.

Results: BPI average pain decreased over time (p=0.005), but treatment differences were small (Cohen d −0.15 at day 7 to 0.16 at day 49). BPI pain-interference contrasts were not significant at individual time points (all p>0.12). PROMIS sleep disturbance was higher with active cannabis at all estimated time points (all p<0.05; treatment-by-time p=0.002). FACT/GOG-NTX neuropathy ratings favored placebo across 8 weeks (p=0.035; d=0.73), and functional well-being favored placebo at weeks 6–8 (all p<0.02; d −0.78 to −0.94).

Harms and cautions: no severe adverse events; daytime sedation was reported by three active participants, while two placebo participants reported forgetfulness or acid reflux. No misuse signal was reported. The pilot was underpowered, no effect CIs were reported, late daily missingness was present, and no channel-engagement, axonal-protection or chronic-prevention inference is justified. The article abstract describes pain-interference worsening, whereas individual main-text time-point tests were all non-significant; this source-level discrepancy is retained rather than reconciled.

Sources: https://pubmed.ncbi.nlm.nih.gov/40611810/; https://journals.sagepub.com/doi/10.1089/can.2025.0028 Shared reference: [60].

Nielsen et al. 2022

DOI 10.1007/s00520-022-07312-y; main reference [62]. Access: supplied 11-page publisher PDF inspected; full article, Figure 1 and Tables 1–3 read. Public registry record NCT04582591 was located for CINCAN-2, but the article/PubMed display separately prints "NCT 04,167,319"; the registry-identifier discrepancy is unresolved. The journal supplementary file and complete dated protocol/SAP were not independently retrieved; no registry identity is treated as definitive. Registry: https://clinicaltrials.gov/study/NCT04582591; mirror: https://ichgcp.net/clinical-trials-registry/NCT04582591.

Design: Prospective CBD cohort compared with a similar non-CBD cohort; not randomized

Population: First CAPOX or carboplatin/paclitaxel cycle

Intervention: CBD oil 150 mg twice daily for 8 days, beginning one day before chemotherapy

Numbers: CINCAN-2 recruited 54 CBD-treated patients (25 Carbo-Tax; 29 CAPOX). Historical CINCAN-1 controls included 15 CAPOX and 16 Carbo-Tax, with 12 fully evaluable controls in each regimen for both primary modalities.

Primary: adjusted day-4 multi-frequency vibrometry at Dig-II 250 Hz favored CBD in CAPOX (−1.22, 95% CI −2.05 to −0.39) and carboplatin–paclitaxel (−1.76, 95% CI −2.52 to −1.02). CAPOX cold-sensitivity patient-reported items favored CBD; carboplatin–paclitaxel patient-reported outcomes did not show significant differences.

Harms and cautions: mild dizziness, nausea, hypersomnia and stomach pain were reported; stomach pain was considered probably related to CBD/capecitabine. Open-label, non-randomized historical comparison with age, comorbidity, temperature, G-CSF and missingness differences; acute first-cycle outcomes only. This is hypothesis-generating, not causal prevention or channel engagement.

Sources: https://link.springer.com/article/10.1007/s00520-022-07312-y Shared reference: [62].

Hershman et al. 2013; SWOG S0715

DOI 10.1200/JCO.2012.44.8738; main reference [55]. Access: Complete original article PDF in institutional repository read; source PDF includes one repository cover page before the eight-page article.

Design: Randomized double-blind multicentre placebo-controlled parallel-group prevention trial, 24 weeks

Population: Women with stage I-III breast cancer starting adjuvant taxane chemotherapy; prevention, not treatment of established CIPN

Intervention: Acetyl-L-carnitine 3000 mg/day versus matching placebo for 24 weeks, initiated with taxane therapy

Numbers: accrued: 437; ineligible: 27; eligible: 410; withdrew all followup consent: 1; evaluable: 409; evaluable active placebo: 208; 201; week 12 FACT NTX observed active placebo: 191; 181; week 24 FACT NTX observed active placebo: 174; 173; toxicity evaluable active placebo: 201; 194; initial ALC placebo among 437 registered: 219; 218; initial arm source: 2018 same-cohort follow-up Figure 1, DOI 10.1093/jnci/djx259; not independent replication; ineligible ALC placebo: 10; 17; ineligible reasons: incomplete baseline information ALC placebo: 4; 10; no baseline blood ALC placebo: 5; 6; prior taxane ALC placebo: 1; 1; additional consent withdrawal ALC placebo: 1; 0; week 12 visit eligible 2018 flow: 192; 182; week 24 visit eligible 2018 flow: 180; 176; safety table 4 ALC placebo: 202; 194; safety denominator discrepancy: 2013 Figure 1 active toxicity n = 201 versus Table 4 n = 202; placebo n = 194 in both. Do not derive a rate by silently choosing a denominator. Shared reference: [58].

Primary: outcome: Baseline-to-week 12 FACT-NTX comparison; higher score indicates fewer neuropathy symptoms; time: 12 weeks; reported model: Week 12 FACT-NTX score adjusted for baseline score, planned taxane regimen and age; adjusted difference active minus placebo: -0.91; 95% CI: -2.21; 0.39; p: 0.17; additional change score model p: 0.23; finding: No demonstrated preventive benefit at the prespecified primary time point; scale: 11-item FACT-NTX, 0–44; lower score means worse symptoms. Current registry 0–64 is discrepant.

Secondary: outcome: Week 24 FACT-NTX score, adjusted model; time: 24 weeks; adjusted difference active minus placebo: -1.8; 95% CI: -3.16; -0.43; p: 0.01; additional change score model p: 0.02; finding: Worse neuropathy score with acetyl-L-carnitine

Function: outcome: FACT-Taxane Trial Outcome Index at week 24, adjusted model; adjusted difference active minus placebo: -3.5; 95% CI: -6.5; -0.4; p: 0.03; interpretation: Worse functional status

Harms: Neuropathy worsening is a clinically relevant adverse finding; do not present the study merely as neutral because its week 12 primary test was nonsignificant

Cautions: Week 12 is primary and week 24 secondary; do not merge their conclusions or interchange time-specific denominators; Reported adjusted-score model P values differ from additional change-score models; retain model labels; Later follow-up is the same cohort, not independent replication; findings do not establish effects of other formulations or treatment of established CIPN; The 2018 original flow diagram resolves initial assigned arms 219/218 and the group-specific exclusions; 409 is the resulting evaluable cohort, not the initial registered population; The 2013 effect-specific FACT-NTX denominators are 191/181 at week 12 and 174/173 at week 24. The later 192/182 and 180/176 visit-evaluable counts and medication-form counts must not replace them; The 2018 week-24 reanalysis is −1.68 (95% CI −3.02 to −0.33; P = .02), distinct from the 2013 Table 3 target result. The later ≤5% missing statement has an unresolved denominator and does not establish low attrition from 409; its 10-point sensitivity changes the endpoint threshold, not missing-data assumptions; The 2013 Table 4 P = .46 compares grade ≥1 neuropathy, not specifically the 8 versus 1 grade-3/4 contrast. No severe-event CI is reconstructed from these source summaries.

Appraisal: the specified result is assessed in Table S4 and Data S1.

Source locators: design dose and primary definition: PDF page 3 (article page 2): Patients and Methods, Study Design, Outcome Measures, Statistical Considerations; flow and week 12 denominators result: PDF page 3 (article page 2): Results, Ac-

crual Eligibility and Evaluability; FACT-NTX Subscale Score; flow figure and week 24 denominators result: PDF page 4 (article page 3): Figure 1; FACT-NTX Subscale Score continuation; function: PDF page 5 (article page 4): Functional Status; effect models: Table 3 plus Results FACT-NTX text; primary/secondary timing defined in Methods; followup flow: 2018 Figure 1 and three-page supplementary figures, checked separately from 2013 outcome Table 3

Registry source: https://clinicaltrials.gov/study/NCT00775645

Sources: https://escholarship.org/content/qt4ph1164g/qt4ph1164g.pdf; https://doi.org/10.1200/JCO.2012.44.8738; https://pubmed.ncbi.nlm.nih.gov/23733756/; https://academic.oup.com/jnci/article/110/6/669/4816962; https://clinicaltrials.gov/study/NCT00775645 Shared reference: [55].

Lux et al. 2024; QUCIP

DOI 10.3389/fonc.2024.1452099; main reference [30]. Access: Complete Frontiers HTML/PDF; Figure 1 and Table 2 visually inspected. Official DRKS English/German records read and archived. Full SAP not available from these public sources.

Design: Prospective multicentre single-arm observational study, 36 weeks

Population: Women with breast-cancer-related painful CIPN

Intervention: High-concentration capsaicin patch, 179 mg; up to three treatments

Numbers: treated: 73; completed 36 weeks: 38; discontinued: 35; primary observed baseline week 12: 72; 56; week 12 any visit data: 58; week 36 any visit data: 40

Primary: outcome: painDETECT final score at week 12 versus baseline; means: 19.71; 15.8; reported paired change: -4.0; SD change: 6.05; p: <0.0001; 95% CI: NR; observed n baseline week 12: 72; 56; paired change n: Not separately stated in Table 2; do not reconstruct CI by assuming that marginal week 12 n = 56 is the complete paired-data n.

Secondary: NPRS 24h baseline week 12: 6.23; 5.01; observed n: 71; 58; reported paired change: -1.21; SD change: 2.08

Harms: 22/73 patients had 34 ADR events; 14/34 events were severe, not 14 severe-event patients. Two serious ADR events (application-site pain and erythema) occurred in one patient. Do not infer comparative safety from this uncontrolled cohort.

Cautions: painDETECT change is not interchangeable with pain intensity and is not validated for longitudinal responsiveness; No causal analgesic, neuroregenerative, or superiority inference; Week 12 painDETECT final-score n = 56 is not the 58 participants with any data at the week 12 visit; primary baseline n = 72 is not the 73-person treated cohort; Thirty-five discontinued over 36 weeks; this is not the number missing the primary week 12 endpoint. Both 36-week retention and endpoint-specific denominators must be labelled; DRKS prospectively identifies painDETECT as primary, but the displayed primary-entry text does not isolate week 12/final score or give a complete SAP. German wording mentions einseitige T-Tests; English wording says one-sample t-tests; paper specifies two-sided tests. The difference does not by itself prove result-driven selection; Paper Section 2.4 describes observed-case and completer analyses, despite referring to an intention-to-treat set. Do not equate the 73-person cohort with complete imputed primary follow-up; Concomitant therapy was recorded, but Discussion states increasing missingness and insufficient documentation of dose changes. Similar care before and after the intervention is not established; Validation for detecting neuropathic pain, longitudinal responsiveness, and study-specific reliability are distinct issues. The authors question longitudinal validation; item 8 must not conflate that with reliability.

Appraisal: the specified result is assessed in Table S4 and Data S1

Source locators: design and primary: PDF pp. 3–4: Section 2.1–2.2; Figure 1 p4; analysis and missingness: PDF p. 5: Section 2.4; Section 3.1 pp. 5–6; Table 2 p7; outcome details: Table 2 p7; Section 3.2 p6; Figure 2 p7; concomitant care: Section 2.2 p4; Section 3.3 p9; Discussion p11; safety: Section 3.5 p9; registry: DRKS00022442: Organizational Data; Endpoints; Recruitment period; Study protocol and further study documents

Registry source: DRKS00022442; prospectively registered 28 July 2020; recorded actual start 20 August 2020; current record last updated 20 August 2021; target 77, no final sample entered

Sources: https://www.frontiersin.org/journals/oncology/articles/10.3389/fonc.2024.1452099/full; https://drks.de/search/en/trial/DRKS00022442; https://drks.de/search/de/trial/DRKS00022442

Table S4 Result specific appraisal and source limits

The eight result-specific worksheets and the QUCIP JBI worksheet do not cover every contextual result listed in Table 2; rows without a worksheet are marked context-only and are not used for cross-study causal comparison.

The scope comprises nine specified results from eight selected studies: eight randomized results and one uncontrolled observation. Hershman contributes distinct 12-week and 24-week results from the same trial. These administrative counts describe this appendix, not systematic coverage of the CIPN literature. Context-only records in S3 are not silently assigned an appraisal.

The parallel-group RoB 2 tool (22 August 2019) is used for Smith, D'Andre and Rao first-period comparisons and the parallel prevention and cannabinoid trials. The crossover tool (18 March 2021) is used for Hincker's both-period secondary pain comparison. First-period selection is still considered in Domain 5. The prior alternative D'Andre form is not counted as another assessment. JBI's accessible 2020 nine-item checklist is explicitly identified; it is not presented as the revised 2024 instrument. Shared references: [8,9,10].

Study	Specific result	Overall RoB 2	Domain judgments
Smith et al. 2013 (CALGB/Alliance 170601)	BPI-SF average-pain change during first 5-week treatment period, assessed on day 1 of weeks 1 and 6; not the second-period or responder result.	Some concerns	D1 Low, D2 Some concerns, D3 Low, D4 Low, D5 Some concerns
D'Andre et al. 2024 (NCT05388058)	EORTC QLQ-CIPN20 sensory-subscale change from baseline to week 2: first randomized period of a two-period crossover trial.	High	D1 Low, D2 Low, D3 High, D4 Low, D5 Some concerns
Weiss et al. 2025 (NCT04398446)	EORTC QLQ-CIPN20 sensory-subscale change baseline to week 12, primary complete-case comparison (23 active/20 placebo), not adjusted longitudinal exploratory model.	High	D1 Low, D2 Low, D3 High, D4 Low, D5 Some concerns
Zimmerman et al. 2016, MC11C4 (NCT01611155)	Published primary EORTC QLQ-CIPN20 sensory-subscale longitudinal comparison during FOLFOX chemotherapy; not the current registry cycle-1 comparison or post-chemotherapy follow-up trajectory.	High	D1 Some concerns, D2 Some concerns, D3 High, D4 Low, D5 Some concerns
Hershman et al. 2013, SWOG S0715 (NCT00775645)	Primary 12-week FACT-NTX neurotoxicity result, linear regression adjusted for baseline FACT-NTX, planned taxane regimen and age stratum; not the 24-week secondary or ≥5-point responder result.	High	D1 Some concerns, D2 Low, D3 High, D4 Low, D5 Some concerns
Hershman et al. 2013, SWOG S0715 (NCT00775645)	Separate main secondary 24-week FACT-NTX adjusted neurotoxicity result; not the 12-week primary result or ≥5-point responder analysis.	High	D1 Some concerns, D2 Low, D3 High, D4 Low, D5 Some concerns
Rao et al. 2007 N00C3; gabapentin versus placebo	First-period week-6 average daily NRS pain comparison, P=0.8; primary efficacy decision point specified in Statistical Considerations; not the week-14 comparison or a pooled crossover estimate	High	D1 Some concerns, D2 Low, D3 High, D4 Low, D5 Some concerns
Hincker et al. 2019; pregabalin versus placebo	Secondary absolute average daily NRS pain reduction over both 28-day treatment periods; paired comparison P = .08. Exact published paired-analysis N remains unresolved across flow, Table 2, Discussion and registry.	High	D1 Low, DS Low, D2 Low, D3 High, D4 High, D5 Some concerns

D1, randomization; DS, period/carryover effects; D2, deviations from intended interventions; D3, missing outcome data; D4, outcome measurement; D5, selection of the reported result. These are RoB 2 judgments about a specified estimate, not intervention-level confidence categories or scores. No assessor override is applied in the current worksheets. Some High outputs follow a conditional route containing NI; this means unresolved risk, not demonstrated distortion or misconduct.

* Smith D3: eAppendix now directly verifies the reported multiple-imputation and control-based pattern-mixture sensitivity analyses. Low is the algorithm output for the specified result; the complete prospective SAP and registry–article analysis correspondence remain unresolved, but the sensitivity methods are no longer classified as NOT_ACCESSED.

Item-level answers, evidence, source locators and tool versions are supplied in Data S1.

Smith et al. 2013 (CALGB/Alliance 170601)

Result assessed: BPI-SF average-pain change during first 5-week treatment period, assessed on day 1 of weeks 1 and 6; not the second-period or responder result.

Overall: Some concerns. Some concerns arise from excluding treatment nonstarters and the unresolved prospective analysis-plan/registry-analysis correspondence. The missing-data domain is supported by the verified eAppendix sensitivity analyses; their methods, imputed contrasts and week-specific imputation proportions are now source-checked. This appraisal does not invalidate or certify the clinical effect.

Observed primary outcomes remain 181 (87/94), distinct from 231 randomized and 220 treatment starters. Public reports contain conflicting no-data, completion and toxicity labels, which were not reconciled by subtraction.

The supplied eAppendix reports multiple imputation using 100 MCMC-based datasets followed by regression imputation and a control-based pattern-mixture model. The imputed between-group differences were 0.750 (p=.0015) and 0.667 (p=.0037); week-specific imputation proportions were 7.1%, 10.8%, 13.7%, 14.6% and 14.6% for weeks 2–6. These details support the D3 assessment, while the imputed analysis population and a dated complete prospective SAP remain unreported or unverified.

The current registry identifies a Wilcoxon comparison whereas the article describes baseline-adjusted ANCOVA. A dated complete prospective SAP was not obtained. This is an unresolved correspondence issue, not proof of significance-driven selection.

D'Andre et al. 2024 (NCT05388058)

Result assessed: EORTC QLQ-CIPN20 sensory-subscale change from baseline to week 2: first randomized period of a two-period crossover trial.

Overall: High. The specified first-period result has a default High missing-data output: 3.1 N, 3.2 PN, 3.3 PY, 3.4 NI. Underlying reasons for unavailable observations are not reported. High denotes unresolved risk under this conditional algorithm, not proof that the estimate was distorted; D5 also has some concerns.

Original Figures 1–2 verify 35 primary observations (19 CBD-first / 16 placebo-first), not the registry's common 20/17 series denominator. One ineligible participant, cancellations and absent observations are flow categories; their underlying reasons are not inferred.

The available protocol includes mixed models, so their use is not treated as an analysis departure. A dated prospective lock and the reported primary between-group mean-change CI remain unverified.

Protocol dosing is 8 mg/day for hands or feet only and 16 mg/day for both, using the specially manufactured CBD-isolate cream. The protocol states no formal PK study for this product; this does not establish that no related-product PK evidence exists.

Weiss et al. 2025 (NCT04398446)

Result assessed: EORTC QLQ-CIPN20 sensory-subscale change baseline to week 12, primary complete-case comparison (23 active/20 placebo), not adjusted longitudinal exploratory model.

Overall: High. High follows the unresolved missing-data pathway, with additional concerns about the prospective outcome/analysis plan. The null primary complete-case result is not replaced by the significant exploratory adjusted model.

Randomized 24/22 and observed week 12 primary 23/20 are confirmed. The three missing outcomes have no reported reason. The eight withdrawals shown before randomization are not used to explain them.

Original Figure 1 gives 98 eligible whereas the narrative/abstract gives 124; these screening counts are retained as a source discrepancy and do not replace the randomized or analysis populations. Registry enrollment 56 is another differently labeled count, not the primary denominator.

The complete supplement does not supply MNAR sensitivity analysis or a locked SAP. Its time-since-chemotherapy analyses and the all 46 exploratory mixed model do not establish missingness robustness for the 43-person primary contrast.

Zimmerman et al. 2016, MC11C4 (NCT01611155)

Result assessed: Published primary EORTC QLQ-CIPN20 sensory-subscale longitudinal comparison during FOLFOX chemotherapy; not the current registry cycle-1 comparison or post-chemotherapy follow-up trajectory.

Overall: High. High follows unresolved missing outcome data (N/PN/PY/NI), with concerns about concealment, initial analysis exclusions and prospective result selection. No observed neuropathy-driven missingness mechanism or significance-driven outcome switch is asserted.

The longitudinal sensory result remains P = .55; the current registry cycle-1 comparison P = .87 is a different result and is not substituted. No numerical longitudinal effect coefficient or confidence interval was identified.

Figure 1 gives 43 primary-evaluable participants (22/21), whereas the current registry labels 16/18 as completed/primary-evaluable. The main-text treated count 24/24 also differs from Figure 1 and registry 25/25. Cycle-specific counts vary and are not summed or equated to patient dropout.

An original Alliance compliance report (Atherton et al., 2016; DOI 10.1177/1740774516655101, Table 3) reports 498 of 543 expected MC11C4 questionnaire booklets returned (91.7%; 47 persons). Booklet-level compliance is not arm-specific primary-outcome completeness or a missing-not-at-random sensitivity analysis. Its denominators do not replace the trial population. Shared reference: [56].

Full dated analysis-plan locking, allocation-concealment procedures and participant-by-time reasons for missing sensory outcomes remain unverified.

Hershman et al. 2013, SWOG S0715 (NCT00775645)

Result assessed: Primary 12-week FACT-NTX neurotoxicity result, linear regression adjusted for baseline FACT-NTX, planned taxane regimen and age stratum; not the 24-week secondary or ≥5-point responder result.

Overall: High. High for the 12-week primary result follows N/PN/PY/NI in the missing-data domain; the mechanism remains unknown rather than demonstrated to be outcome dependent. Concealment and prospective analysis locking also remain incompletely documented. The 24-week adverse result is not promoted to the primary endpoint.

The original 2018 follow-up Figure 1 (DOI 10.1093/jnci/djx259) resolves the initial arms among 437 registered participants: 219 acetyl-L-carnitine and 218 placebo. Ineligible participants numbered 10/17, with one additional acetyl-L-carnitine consent withdrawal, leaving 208/201 evaluable. Shared reference: [58].

For the 2013 estimates, observed FACT-NTX counts were 191/181 at week 12 and 174/173 at week 24. The later flow diagram instead lists 192/182 and 180/176 eligible for evaluation at those visits; these are not interchangeable endpoint denominators.

The 2013 Table 3 adjusted effects are −0.91 (95% CI −2.21 to 0.39; P = .17) at week 12 and −1.80 (−3.16 to −0.43; P = .01) at week 24. The 2018 week-24 reanalysis −1.68 (−3.02 to −0.33; P = .02) is a different analysis, not replacement data or independent replication.

The later report's statement of no more than 5% missing at each time point does not specify a denominator that resolves loss from the initial/evaluable cohort. Its 10-point threshold sensitivity concerns endpoint definition, not missing-data assumptions. No person-specific missingness explanations or dated locked SAP were obtained.

FACT-NTX has 11 items with a 0–44 range in the original follow-up, not the current registry's 0–64. In the 2013 safety table, P = .46 concerns grade ≥1 neuropathy, not the 8 versus 1 severe-neuropathy contrast. Table 4 and Figure 1 differ by one in the active safety denominator.

Hershman et al. 2013, SWOG S0715 (NCT00775645)

Result assessed: Separate main secondary 24-week FACT-NTX adjusted neurotoxicity result; not the 12-week primary result or ≥5-point responder analysis.

Overall: High. High for the 24-week secondary result follows N/PN/PY/NI in the missing-data domain; the mechanism remains unknown rather than demonstrated to be outcome dependent. Concealment and prospective analysis locking also remain incompletely documented. The 24-week adverse result is not promoted to the primary endpoint.

The participant flow, observed denominators, original and follow-up estimates, and common source limitations are detailed in the preceding 12-week entry. This is a separate result-specific assessment of the 24-week secondary outcome, not an independent trial or replication.

Rao et al. 2007 N00C3; gabapentin versus placebo

Result assessed: First-period week-6 average daily NRS pain comparison, P=0.8; primary efficacy decision point specified in Statistical Considerations; not the week-14 comparison or a pooled crossover estimate

Overall: High. High follows substantial missing week-6 outcomes and unresolved outcome dependence (N/PN/PY/NI). The first-period selection is explicit in the paper, but concealment and a locked prospective plan remain unverified. No complete-ITT, pharmacological carryover or proven selective-reporting claim is made.

The parallel RoB 2 form evaluates the first-period result, consistently with the Smith and D'Andre first-period comparisons. Selection of the reported first-period result is considered in Domain 5.

NCT00027963 is explicitly linked by NCCTG-N00C3 and the article identifier. Its enrollment of 100 differs from the published 115; the published analysis retains its own denominator. Table 2 placebo NRS is 3.0 whereas prose gives 3.1; no precise derived effect or CI is reconstructed.

Safety Table 3 uses exposure counts 91/89, not the sequence counts 57/58. Figure 2 is now visually verified and reports the week-6 and week-14 flow counts; individual first-period withdrawal reasons remain unreported. Generic robustness language is not treated as a complete missing-data sensitivity analysis.

Hincker et al. 2019; pregabalin versus placebo

Result assessed: Secondary absolute average daily NRS pain reduction over both 28-day treatment periods; paired comparison P = .08. Exact published paired-analysis N remains unresolved across flow, Table 2, Discussion and registry.

Overall: High. High follows the missing-data domain and the tool's conditional measurement-bias pathway. The source audit now verifies the protocol and sequence allocation, but neither the exact published paired N nor actual influence of allocation awareness is established. This is a secondary pain result, not the trial's primary prediction test.

The official protocol/SAP was obtained. The planned blinding question is day 5 of period 1 on p. 13, with inconsistent generic wording on p. 10. The actual date and prospective version lock remain unverified.

Sequence counts are 12/14; first-period completion and second-period initiation are 11/13; second-period completion is 11/9. Twenty-four entered both periods, 23 had the primary QST predictor data, and Table 2 headers give 25 per treatment. These are not a verified common paired-analysis denominator.

Published average-pain reductions are 0.8 and 0.5 points (both SD 1.1; P = .08). Registry changes are −1.0 and +0.3 (n = 24 each). Direction labels alone do not reconcile the estimates, and no paired CI is derived. The placebo responder entry 3 (8%) is also internally inconsistent.

The primary predictor average-pain P value is .92 in Results but .97 in Abstract/Discussion. Neither is the secondary average-pain P = .08. Dose reductions and some withdrawals were permitted by the protocol; their occurrence is not automatically a protocol violation.

QUCIP single arm observation

JBI quasi-experimental checklist, 2020 version. Target: baseline-to-week-12 painDETECT change. The checklist is not converted to a numerical score, RoB 2 category or certainty grade. Shared reference: [10].

Item topic	Answer	Evidence and boundary
1. Temporal order	Yes	Baseline preceded patch treatment and visit 1 follow-up. Identifiable temporal order alone does not establish causation.
2. Same participants in compared observations	Yes	Within-person pre/post comparison. The 2020 guidance permits the same participants; loss of follow-up is assessed separately in item 6.
3. Similar care apart from intervention	Unclear	Concomitant medication and nonpharmacological care were recorded, but dose changes could not be assessed reliably. Unchanged other care is not established.
4. Independent control group	No	Prospective single-arm observation without untreated or sham comparison. Baseline is not an independent control group.
5. Multiple observations before and after	No	One pretreatment baseline and several post-treatment visits; no repeated pretreatment measures. Repeated follow-up alone does not satisfy this item.

Item topic	Answer	Evidence and boundary
6. Follow-up and handling of incomplete data	No	Among 73 treated, primary final-score observations number 72 at baseline and 56 at week 12; 58 had some week-12 data. Observed-case analysis does not adequately address the effect of missingness. Thirty-five discontinuations occurred over all 36 weeks, not necessarily before week 12.
7. Consistent outcome measurement	Yes	The same painDETECT final score was used at the compared times. Its 0–38 final score, 0–35 total symptom score and NPRS are distinct endpoints.
8. Reliable measurement in this study	Unclear	Instrument background is described, but study-specific reliability/repeatability evidence is not verified. The authors' separate longitudinal-validity concern is not misrepresented as evidence of poor reliability.
9. Appropriate statistical analysis	Unclear	Paired-change tests can describe observed within-person change, but exact paired N, distributional checks and full missing-data handling are unverified. A full SAP is unavailable, and German/English registry test descriptions differ from one another and the paper. The earlier broadly qualified Yes is narrowed to Unclear; this does not prove the test was invalid or result-selected.

DRKS00022442 was registered on 28 July 2020 before the recorded actual start on 20 August 2020. It identifies painDETECT, but the unique week-12 final-score specification and complete SAP are not verified. Its stale recruitment status is not treated as current study status.

Primary marginal means are 19.71 (SD 4.69; n = 72) at baseline and 15.80 (SD 6.20; n = 56) at week 12. Reported paired change is −4.00 (SD 6.05; $P < .0001$), with paired-change N not separately labelled. No CI is back-calculated from an assumed paired N.

Adverse reactions affected 22/73 participants (34 events); 14 events were severe, and two serious events occurred in one participant. Event counts are not numbers of affected people. Thirty-eight completed 36 weeks, whereas 40 supplied some visit-3 data.

Item 9 is now Unclear, not a numerical downgrade or proof of erroneous statistics. No total quality score, RoB 2 category or cross-product confidence grade is calculated.

Disposition: descriptive human evidence only. The absence of a concurrent control and unresolved concomitant care and missingness prevent causal efficacy inference. Full source locators and the public registration record are in Data S1. The use of structured tools does not convert this narrative review into a systematic review or remedy the lack of independent duplicate assessment.

Table S5 Defined-product six-domain examples

Source-linked reporting examples, not a score or candidate ranking.

Defined natural-product example	Evidence setting	Translational stage and unresolved link
Isolated cannabidiol	Human Kv7.2/Kv7.3 expression assay	Not a chemotherapy assay; a clinical formulation cannot inherit this mechanism without a matched exposure and engagement bridge.
Isolated harmaline	Recombinant human NaV1.7 assay	No demonstrated human CIPN exposure, efficacy or therapeutic window in the cited study.
Narirutin	Channel assay and non-CIPN nerve-injury experiment	Not a chemotherapy model; neither human CIPN efficacy nor achievable human unbound exposure is established.
Capsaicin 8% patch	Human non-interventional CIPN cohort	Defined clinical product, but uncontrolled symptom observations do not demonstrate comparative efficacy, channel engagement or axonal preservation.

The rows summarize evidence settings and are not ordinal stages; no candidate ranking is assigned. The detailed source-linked fields follow. Suzetrigine is listed afterward only as a non-natural-product clinical comparator.

Isolated cannabidiol in a channel assay

Subtype: Human Kv7.2/Kv7.3; activation-voltage shift

Potency: EC50 214 nM for the voltage shift; not a CIPN therapeutic threshold

Exposure: No matched unbound nerve exposure measured in this assay. Licensed oral cannabidiol is >94% protein-bound in vitro; its label cannot establish exposure from a CIPN cream or THC mixture.

Selectivity: This channel observation is not a comprehensive off-target panel; cannabidiol has additional pharmacology.

CIPN: No chemotherapy in the cited channel assay. Separate CIPN trials concern non-equivalent clinical formulations.

Safety: No exposure-based CIPN safety margin established by the assay. Oral-label interactions and hepatic warnings remain product/context dependent.

Source location: Zhang 2022, Figure 1; EPIDIOLEX prescribing information, sections 5, 7 and 12.3. https://elifesciences.org/articles/73246; https://dailymed.nlm.nih.gov/dailymed/fda/fdaDrugXsl.cfm?setid=8bf27097-4870-43fb-94f0-f3d0871d1eec Shared references: [34,68].

Isolated harmaline

Subtype: Recombinant human NaV1.7 in CHO cells; inhibition

Potency: IC50 35.5 μM; automated patch clamp

Exposure: Achievable unbound peripheral exposure was not established in the channel paper; no clinical exposure-to-potency ratio calculated.

Selectivity: Limited subtype comparisons, not a full human channel safety panel.

CIPN: No chemotherapy-specific model in this channel study.

Safety: Central nervous system and tremor liabilities; no demonstrated CIPN therapeutic window.

Source location: Eisfeld 2025, Results and Discussion. https://pmc.ncbi.nlm.nih.gov/articles/PMC12111501/ Shared reference: [66].

Narirutin in a non-CIPN experimental system

Subtype: Mouse NaV1.7 expressed in HEK293 cells; current inhibition

Potency: 20 μM overnight reduced peak NaV1.7 current density by 52.78%; this single-concentration result is not an IC50.

Exposure: Rat intrathecal administration in spared-nerve injury, 20 μg/20 μL; not human unbound exposure or an oral formulation.

Selectivity: Pharmacological interaction with a NaV1.7 inhibitor does not establish broad off-target selectivity.

CIPN: Spared-nerve injury, not chemotherapy-induced neuropathy.

Safety: No human CIPN exposure-based margin or oncology compatibility established in the cited report.

Source location: Yang 2022, Methods and channel-current Results. https://pmc.ncbi.nlm.nih.gov/articles/PMC9738487/ Shared reference: [65].

Capsaicin 8% cutaneous patch

Subtype: TRPV1 agonism followed by local nociceptor defunctionalization

Potency: 179 mg per 280 cm^2 patch; product strength is not a channel EC50.

Exposure: Low, transient systemic exposure in label PK studies; unbound concentration at skin nerve endings not provided. Plasma PK from other neuropathies does not quantify CIPN target coverage.

Selectivity: TRPV1 pharmacology; a labeled mechanism is not a patient-specific engagement measurement.

CIPN: QUCIP is a prospective non-interventional CIPN cohort, not a randomized efficacy test.

Safety: Local pain, burns and sensory changes require assessment. No dose-to-potency-derived CIPN safety window established.

Source location: Lux 2024; Qutenza SmPC sections 2, 4.4, 5.1 and 5.2. https://doi.org/10.3389/fonc.2024.1452099; https://www.medicines.org.uk/emc/product/573/smpc Shared reference: [30]. Shared reference: [31].

Suzetrigine as an external clinical precedent

Subtype: Human NaV1.8 inhibition

Potency: IC50 0.68 nM in human DRG experiments; species-dependent potency

Exposure: Approved acute-pain dosing does not establish unbound exposure or sustained engagement in chemotherapy-exposed nerves.

Selectivity: Strong selectivity over other human NaV subtypes in the cited pharmacology study.

CIPN: No CIPN indication established by the FDA label or acute-pain trials.

Safety: CYP3A interaction restrictions; treatment beyond 14 days not studied in the label. Long-term oncology compatibility cannot be inferred.

Source location: Osteen 2025; JOURNAVX January 2026 label. https://pmc.ncbi.nlm.nih.gov/articles/PMC11914629/; https://www.accessdata.fda.gov/drugsatfda_docs/label/2026/219209s009lbl.pdf Shared references: [25,26].

Table S6 Botanical identity reporting requirements

Required field	Information needed
Identity	Authenticated Latin binomial, plant part, voucher or equivalent authentication; distinguish species and preparation.
Preparation	Extraction solvent, procedure, ratio and formulation; distinguish extract mass from raw-herb equivalent.
Composition	Chromatographic fingerprint and marker compounds with analytical methods; a marker is not automatically the active constituent.
Manufacturing	Manufacturer, batch/lot, quality controls, stability and contamination testing; document comparability across experiments.
Administration	Dose units, route, frequency, timing relative to chemotherapy and duration.
Mechanistic bridge	Link the same batch/product to channel assay, relevant active exposure and CIPN system.
Safety context	Product-specific toxicology, interactions and oncology compatibility; do not transfer evidence from a related formulation.

This is a reporting template, not a completed characterization of a herbal intervention. Supporting framework: MIABE, DOI 10.1038/nrd3503; FDA Botanical Drug Development Guidance for Industry (2016). No verified batch identity is invented. Shared references: [11,67].

Table S7 Targeted pharmacokinetic exposure selectivity and safety audit of retained natural product examples

This targeted audit was added after the reviewer-requested six-domain check. It is not a systematic pharmacokinetic or toxicology review. Values are restricted to the cited formulation, route, species and indication. “Not reported” means that the inspected source did not provide the field; it does not mean that the field was never measured. No unbound exposure, therapeutic window or selectivity margin was inferred across products.

Product and source setting	Potency and exposure	Selectivity and CIPN context	Safety and oncology compatibility
Isolated cannabidiol (CBD)<br>Human Kv7.2/Kv7.3 assay	Activation-voltage shift EC50 214 nM. No matched unbound nerve exposure. Licensed oral cannabidiol is >94% protein-bound in vitro and has a repeated-dose plasma half-life of approximately 56–61 h; these values are not transferable to topical CBD or CBD/THC products.	The assay is not a comprehensive off-target panel. No chemotherapy was used in the channel assay; clinical CBD formulations do not establish Kv7 target engagement.	The oral label reports dose-related transaminase elevations and CYP/UGT interaction warnings. No CIPN exposure-based therapeutic or oncology-compatibility window.

Product and source setting	Potency and exposure	Selectivity and CIPN context	Safety and oncology compatibility
CBD-isolate cream D'Andre et al. 2024	Specially manufactured 0% THC cream, twice daily. Protocol dose 8 mg/day for one treated site or 16 mg/day for both. Protocol states that no formal PK study had been performed for this product.	No channel-resolved potency, subtype panel or patient target-engagement measurement. The first-period sensory endpoint did not demonstrate benefit for this cream and dose.	No product-specific exposure-based safety window. Registry harms cover the registry-defined safety period and are not a first-period CBD-causality estimate.
Pure oral CBD oil Nielsen et al. 2022	100 mg/mL GMP product; 150 mg twice daily for 8 days. Reported impurities: CBDV 0.2%, CBD-C4 0.1%, THC <0.01%. The paper discusses approximately 6% oral bioavailability as background pharmacology but reports no participant-specific plasma or unbound concentrations.	No channel assay or product-specific selectivity panel. Prospective nonrandomized historical comparison of acute first-cycle symptoms in CAPOX and carboplatin/paclitaxel cohorts.	Dizziness, stomach pain, hypersomnia and nausea were reported. No product-specific therapeutic or oncology-compatibility window.
Plant-derived oral CBD/THC Weiss et al. 2025	Approximately 125.3–135.9 mg CBD/day plus 6.0–10.8 mg THC/day with minor cannabinoids and terpenes. No product-specific plasma, unbound or nerve-tissue exposure in the article or supplement.	No channel-resolved potency or subtype panel. Primary week-12 complete-case comparison was not significant; the adjusted model was exploratory. This mixture is not interchangeable with purified or topical CBD.	No serious adverse events or AE-related withdrawals reported in the inspected source. No dose-to-potency or oncology-compatibility window.
Oral CBD/THC capsules Haney et al. 2025	100 mg CBD plus 5 mg THC per capsule, up to three capsules/day. No product-specific plasma, unbound or nerve-tissue exposure reported.	No channel-resolved potency or subtype panel. Very small randomized pilot in established painful taxane-related CIPN; no efficacy CI reported.	Sedation occurred in active participants. No exposure-based safety window or oncology-compatibility window.
Capsaicin 8% patch Qutenza and QUCIP	179 mg per 280 cm² patch. After 60-min applications, systemic capsaicin was <5 ng/mL in about one-third of PHN patients, 3% of painful-DPN patients and none of HIV-AN patients; maximum 4.6 ng/mL; population PK using 60-/90-min application data estimated a mean terminal half-life about 130 min. Free concentration at CIPN nerve endings was not measured.	TRPV1 agonism followed by local nociceptor defunctionalization. Product strength is not a TRPV1 EC50; patient target engagement was not measured. QUCIP was noninterventional.	Local application pain, burns, erythema and sensory changes remain relevant. No CIPN dose-to-potency or oncology-interaction window.

Product and source setting	Potency and exposure	Selectivity and CIPN context	Safety and oncology compatibility
Harmaline<br>Recombinant human NaV1.7 assay	Automated patch-clamp IC50 35.5 µM. No human PK or achievable unbound peripheral-nerve exposure.	Limited subtype comparisons; no full sodium-channel/off-target panel. No chemotherapy-specific model or human CIPN study in the inspected source.	CNS and tremor liabilities are relevant; any monoamine-oxidase activity requires independent source support. No CIPN therapeutic or oncology-compatibility window.
Narirutin<br>SNI rat model and NaV1.7 assay	20 µM overnight reduced recombinant NaV1.7 peak-current density by approximately 52.78%; this is not an IC50. In-vivo dose 20 µg/20 µL intrathecally. No human PK or unbound peripheral exposure.	No effect on TTX-resistant current under the cited conditions, but not a full off-target panel. Spared-nerve-injury model, not CIPN.	No human CIPN safety margin, systemic safety window or oncology-compatibility assessment.
Huangqi Guizhi Wuwu Decoction granules<br>Sun et al. 2026	47.5 g sachet twice daily during XELOX. Abstract does not report active-constituent concentrations, human PK, unbound exposure or nerve-tissue concentration. Botanical batch, marker compounds and manufacturing details require the full article or supplement.	Publication-reported abstract values only; registry provenance remains unresolved and these values are retained for traceability, not used in comparative efficacy synthesis: 360 enrolled, 354 mITT; grade ≥2 chronic OIPN 21.3% versus 38.6%; reported OR 0.43 (95% CI 0.27–0.69), P<0.001. No channel subtype or potency data were reported.	Publication-abstract-reported AE OR 1.27 (95% CI 0.68–2.39). Detailed AE categories, oncology compatibility and product-specific safety window remain unverified. Publication cites NCT04913376, which conflicts with an earlier hilotherapy trial using the same identifier; registry details were not substituted.
Acetyl-L-carnitine (ALC)<br>Indirect comparator; SWOG S0715	SWOG S0715 used 3 g/day for 24 weeks. A matched healthy-volunteer PK study used one 500-mg tablet: Tmax 3.1 h, terminal half-life 4.2 h and AUC0–8 9.88 µg·h/mL (n=8). These data do not establish free peripheral-nerve exposure at the chemotherapy dose.	No direct ion-channel potency, subtype selectivity or channel-engagement measurement was reported; ALC remains outside the core channel matrix.	General carnitine sources report gastrointestinal effects and fishy body odour near 3 g/day and caution in seizure disorders and severe renal dysfunction/uremia. No CIPN-specific exposure-to-toxicity or oncology-compatibility window was established. Indirect comparator outside the core channel matrix; no channel-engagement or matched exposure bridge.

Interpretation: These rows are reporting records, not rankings. The natural products and formulations are not ranked; no product has a complete channel potency–unbound exposure–selectivity–CIPN model–safety-window bridge, and missing fields are not interpreted as evidence of absence. The editor-identified correction-linked Huangqi source remains excluded; the Sun et al. 2026 publication is treated as contextual publication-level evidence with unresolved registry provenance.

NEW FULL-TEXT MECHANISTIC ADDENDUM — 3–4 October 2026

WEDEL2026 — TRPV1/18:1 lysophospholipids

Wedel S et al., 2026; DOI 10.1073/pnas.2613615123. Role: core mechanistic update; access: 18-page SAPP retrieved, relevant sections verified; publisher metadata and Zenodo data checked. Human-linked acute lipid–TRPV1 mechanism, not clinical efficacy or patient target engagement. Shared reference: [21].
System/exposure: 35 adults with breast/ovarian cancer from a PrevTel-derived paclitaxel-treated cohort; weekly paclitaxel 80 mg/m^2; plasma day 0/14/15 and day 16/17 time-course. Cryotherapy and concomitant/prior cancer therapy remain confounders.
Source location: main article relevant Methods/Results; SAPP pp3–17, Figures S1–S8 and SI Methods; Zenodo DOI 10.5281/zenodo.19494039.
TSUBAKI2026_LEP4 — oxaliplatin/LEP-4
Tsubaki M et al., 2026; DOI 10.1155/adpp/4435116. Role: boundary formulation example; access: main article full text verified; publisher supplementary tables/figures not independently retrieved. LEP-4 is an unresolved ≤3500-Da fraction, not a purified compound and is not assigned a translational-readiness status. Shared reference: [47].
System/exposure: male BALB/c mice; oxaliplatin 6 mg/kg i.p. days 0/7/14; LEP-4 0.25 g/kg oral daily, including established-allodynia treatment. Measurement: 10 °C cold plate, von Frey, DRG *CACNA1H*/CaV3.2 and NR2A mRNA, DRG/spinal p-ERK1/2. No direct current/gating, composition, PK, selectivity or long-term safety.
Source location: Main article pp2–13, Methods §§2.1–2.10, Results §§3.1–3.7, Figures 1–7; DOI landing page.
DIGIROLAMO2026_NCX2 — exchanger/axon-preservation boundary
Di Girolamo S et al., 2026; DOI 10.3390/cancers18183024. Role: non-channel axonal-preservation comparator; access: main article full text verified; Appendix A/BOARD raw data not independently retrieved. Shared reference: [48].
System/exposure: primary male mouse DRG cultures; oxaliplatin 7.5–50 μM for 24–48 h; SEA0400 1 μM or NCX2/SLC8A2 siRNA. Partial rescue of selected neurite/viability/lipid-droplet endpoints, mainly at low dose. No reverse-mode NCX2, Na^+/Ca^{2+} flux, current, directionality, in vivo or clinical evidence.
Source location: Main article pp3–24, Methods §§2.1–2.7, Results §§3.1–3.6 and Discussion/limitations.
OU2026_METTL3 — central dorsal-horn indirect signal
Ou C et al., 2026; DOI 10.1113/JP291162. Role: indirect central remodeling context; access: full main article relevant sections verified. Shared reference: [49].
System/exposure: male/female C57BL/6J mice; paclitaxel 8 mg/kg i.p. days 0/2/4/6; dorsal-horn slices 24 h after final dose. METTL3–m6A-circMtmr7–STAT5A/NUFIP2 perturbations changed mEPSCs, intrinsic firing and mechanical allodynia. These are synaptic/firing readouts, not ion-channel-resolved currents or target engagement.
Source location: Main article pp3–23, Methods and Figures 1–9; Discussion/limitations pp22–23.
